# Systematic Identification of Characteristic Genes of Ovarian Clear Cell Carcinoma Compared with High-Grade Serous Carcinoma Based on RNA-Sequencing

**DOI:** 10.3390/ijms20184330

**Published:** 2019-09-04

**Authors:** Saya Nagasawa, Kazuhiro Ikeda, Kuniko Horie-Inoue, Sho Sato, Atsuo Itakura, Satoru Takeda, Kosei Hasegawa, Satoshi Inoue

**Affiliations:** 1 Division of Gene Regulation and Signal Transduction, Research Center for Genomic Medicine, Saitama Medical University, Saitama 350-1241, Japan; 2 Department of Obstetrics and Gynecology, Juntendo University, Tokyo 113-8421, Japan; 3 Department of Gynecologic Oncology, Saitama Medical University International Medical Center, Saitama 350-1298, Japan; 4 Department of Systems Aging Science and Medicine, Tokyo Metropolitan Institute of Gerontology Itabashi-ku, Tokyo 173-0015, Japan

**Keywords:** ovarian cancer, gene expression, RNA sequencing, clear cell carcinoma, high-grade serous carcinoma

## Abstract

Objective: Ovarian cancer has the highest mortality among gynecological cancers. High-grade serous carcinoma (HGSC) is the most common histotype of ovarian cancer regardless of ethnicity, whereas clear cell carcinoma (CCC) is more common in East Asians than Caucasians. The elucidation of predominant signaling pathways in these cancers is the first step towards understanding their molecular mechanisms and developing their clinical management. Methods: RNA sequencing was performed for 27 clinical ovarian specimens from Japanese women. Principal component analysis (PCA) was conducted on the sequence data mapped on RefSeq with normalized read counts, and functional annotation analysis was performed on genes with substantial weights in PCA. Knockdown experiments were conducted on the selected genes on the basis of PCA. Results: Functional annotation analysis of PCA-defined genes showed predominant pathways, such as cell growth regulators and blood coagulators in CCC and transcription regulators in HGSC. Knockdown experiments showed that the inhibition of the calcium-dependent protein copine 8 (CPNE8) and the transcription factor basic helix-loop-helix family member e 41 (BHLHE41) repressed the proliferation of CCC- and HGSC-derived cells, respectively. Conclusions: This study identified *CPNE8* and *BHLHE41* as characteristic genes for CCC and HGSC, respectively. The systemic identification of differentially expressed genes in CCC and HGSC will provide useful information to understand transcriptomic differences in these ovarian cancers and to further develop potential diagnostic and therapeutic options for advanced disease.

## 1. Introduction

Ovarian cancer has the highest mortality in gynecological cancers and is responsible for the death of ~4500 women per year in Japan [1]. Because the disease often exhibits no obvious symptoms in the early stage, nearly half of the patients will eventually develop advanced cancer and have a five-year survival rate <30% [2]. High-grade serous carcinoma (HGSC) is the most common histotype among ovarian cancers and is usually diagnosed at an advanced stage exhibiting aggressive behavior [3]. The incidence of HGSC is reported as 70% of malignant ovarian cancer in Caucasians [4]. Interestingly, the nature of ovarian cancer among Japanese patients is rather unique compared to Caucasian patients. Although the incidence of HGSC in Japanese women (35%) is lower than in Caucasian women, that of clear cell carcinoma (CCC) is as high as 24%—being the second highest incidence in Japanese—whereas it is only 10% in Caucasians [5].

Multiple signaling pathways involved in processes such as cellular proliferation, migration, differentiation, and apoptosis have been shown to be associated with the pathophysiology of ovarian cancer. In HGSC, tumorigenesis and therapy resistance are regulated by various signaling pathways including nuclear factor kappa B (NF-κB), Notch, Myc, Wnt/β-Catenin, Hedgehog, phosphatase and tensin homolog (PTEN)/phosphatidylinositol 3-kinase (PI3K)/AKT/mammalian target of rapamycin (mTOR) pathway, mitogen-activated protein kinase (MAPK), insulin-like growth factor (IGF), multi-drug resistance 1 (MDR1), and transforming growth factor β (TGFβ) [6]. For example, immunohistochemical analysis revealed that NF-κB expression, which regulates the expression of proinflammatory genes, correlates with poor prognosis and becomes a risk factor for chemotherapy resistance of serous carcinoma [7]. Notch 3 is implicated in tumor proliferation, and its expression correlates with carboplatin resistance in HGSC [8,9]. Myc is frequently amplified/overexpressed in ovarian HGSC and assumed to function as a key oncogenic driver gene contributing to carcinogenesis [10,11]. IGF signaling pathways are also involved in tumor progression and drug resistance in ovarian cancer. For instance, high IGF2 expression in serous ovarian cancer is significantly associated with a shorter interval to disease progression and death and resistance to the chemotherapy drug Taxol [12,13,14]. MDR1 expression also significantly elevates the risk for ovarian cancer progression [15].

Ovarian CCC mostly arises from endometriosis of the ovary and is commonly detected at a low stage compared with HGSC. Pearce et al. reported that endometriosis is associated with a significant risk of CCC: 20.2% of patients with CCC reported a history of endometriosis [16]. Nevertheless, the overall survival of patients with CCC, with endometrioid cysts in particular, has not been improved by monthly transvaginal echoic examination. Compared with other ovarian cancer histotypes, CCC is often characterized by chemoresistance and high frequency of deep venous thrombosis [17]. Gene expression profiling implies that the overexpression of hepatocyte nuclear factor-1β (HNF-1β) and hypoxia-inducible factor 1 α (HIF-1α) is a key driver event in CCC [18,19].

These findings suggest that different types of ovarian cancer develop along with different molecular pathways. Although there are several reports exploring gene expression profiling specific for these histotypes, new and effective biomarkers have not been applied for clinical use; only few tumor markers including CA125 are recommended in the diagnosis and management of ovarian cancer [20,21,22]. In the 1980s, ovarian cancer prognosis was improved by the emergence of the paclitaxel–carboplatin treatment. Nevertheless, CCC is classified as chemoresistant. In addition, HGSC is initially chemosensitive, but over 60% of these tumors are known to relapse after chemotherapy [1]. Therefore, understanding the histological differences and molecular mechanisms of ovarian cancer progression is essential for the prevention of this cancer and to improve the survival rate of patients. Owing to the low incidence among Caucasians, the molecular mechanisms of ovarian CCC are less characterized than those of HGSC. Considering the different histological incidence between Caucasians and East Asians, we focused on the molecular differences of CCC compared to HGSC.

From the viewpoints described above, we speculated that a particular set of genes associated with distinct ovarian cancer histotypes would contribute to the clinical phenotype of the disease, including tumorigenicity and therapeutic response. To test our hypothesis, clinical specimens from patients with CCC and HGSC, as well as normal ovary specimens, were analyzed by high-throughput RNA sequencing. Functional analysis of candidate genes predominantly expressed in CCC and HGSC revealed distinct signaling pathways in the two cancer histotypes, which would play roles in cancer cell proliferation.

## 2. Results

### 2.1. Principal Component Analysis from RNA-Sequencing Data Reveals Candidate Genes Associated with CCC and HGSC

**Figure 1:**
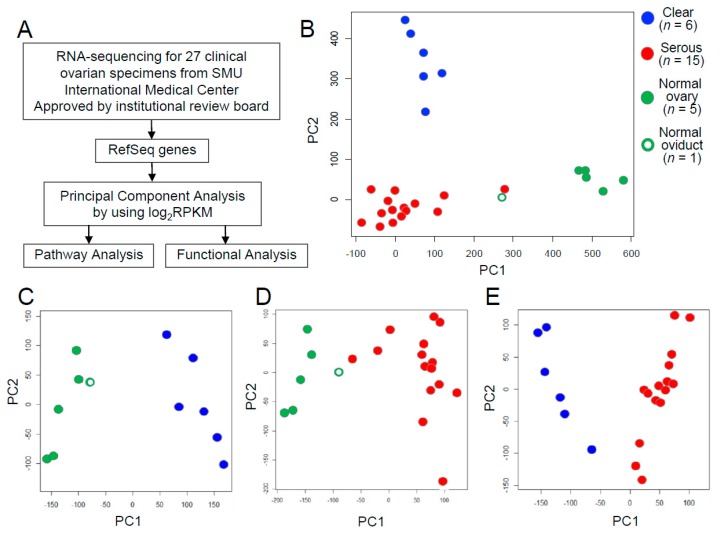
Principal component analysis (PCA) using log_2_ read per kilobase of transcript length per million mapped reads (RPKM) of RefSeq genes showed transcriptomic differences among pathological groups. (**A**) Schematic presentation of transcriptomic and functional analysis of ovarian cancer performed in this study. Briefly, RNAs were isolated from 27 clinical ovarian specimens and analyzed by RNA sequencing. Based on the log2RPKM values for each RefSeq gene, PCA using log2RPKM of Refseq genes was performed. Then, functional analysis using small interfering RNA (siRNA) was also performed. (**B**) Plot of the first two components from PCA of the log2RPKM of RefSeq genes. The prcomp function of R-package was used for the PCA of clear cell carcinoma (CCC) (*n* = 6), high-grade serous carcinoma (HGSC) (*n* = 15), and normal tissues (*n* = 6). PC1, 1st principal component; PC2, 2nd principal component. (**C**) Plot of the first two components of the CCC and normal tissue groups. (**D**) Plot of the first two components of the HGSC and normal tissue groups. (**E**) Plot of the first two components of the CCC and HGSC groups. CCC and HGSC are indicated by blue and red solid circles, respectively. Normal tissues are indicated by green circles, with the solid ones representing normal ovary tissues and the open ones representing normal oviduct tissue.

We performed RNA sequencing of 27 clinical ovarian specimens obtained from patients. Clinical information for the 27 subjects is shown in Supplementary Table S1. To visualize the relationships between the 6 CCCs, 15 HGSCs, and 6 normal tissues based on gene expression, we performed principal component analysis (PCA) based on the expression level represented by log2RPKM value for each RefSeq gene (Figure 1A). The result indicated that the three groups, CCC, HGSC, and normal tissues, could be characterized by the first two principal components: the proportion of the variability regarding the first principal component (PC1) and the second principal component (PC2) was 18.5% and 11.0%, respectively (Figure 1B). One normal tissue sample was derived from the oviduct (green-colored open circle in Figure 1B) and, interestingly, this sample clustered near to the HGSC group, possibly reflecting the characteristics of its developing tissue oviduct from which HGSC would be also generated. We included this normal oviduct as normal tissue because it did not interfere with the main results of the comparison study of the characteristics of CCC and HGSC. Then, we further performed PCA with each pair of these three groups. CCC and HGSC were separated from normal tissues (Figure 1C,D): the proportion of the variability regarding PC1 and PC2 was 36.0% and 12.2% in Figure 1C, respectively, and 23.3% and 9.3% in Figure 1D, respectively. Moreover, we could clearly discriminate between CCC and HGSC (Figure 1E): the proportion of the variability regarding PC1 and PC2 was 14.6% and 10.7%, respectively. These results indicate that differential gene expression profiles of the ovarian cancer histotypes CCC and HGSC are diagnostically important.

To determine the signaling pathways that define the characteristics of these three groups, we performed functional annotation analysis for sets of genes that were defined as contributing genes for each group along PC1 in Figure 1C–E using DAVID Bioinformatics Resources 6.7 (Supplementary Figure S1 and Supplementary Tables S3–S8). Notably, the cell cycle regulation-associated pathway was among the top 10 pathways that were dominant in CCC and HGSC compared with normal tissues (Supplementary Tables S3 and S5). In a comparison between CCC and HGSC, blood coagulation factors and body fluid regulators were specifically annotated as contributing genes separating CCC from HGSC (Supplementary Table S7). These transcriptomic profiles may explain one of the clinical features of patients with ovarian CCC, who are more susceptible to venous thrombosis. On the other hand, various transcription regulators were listed as contributing genes that discriminate HGSC from CCC (Supplementary Table S8).

**Figure 2:**
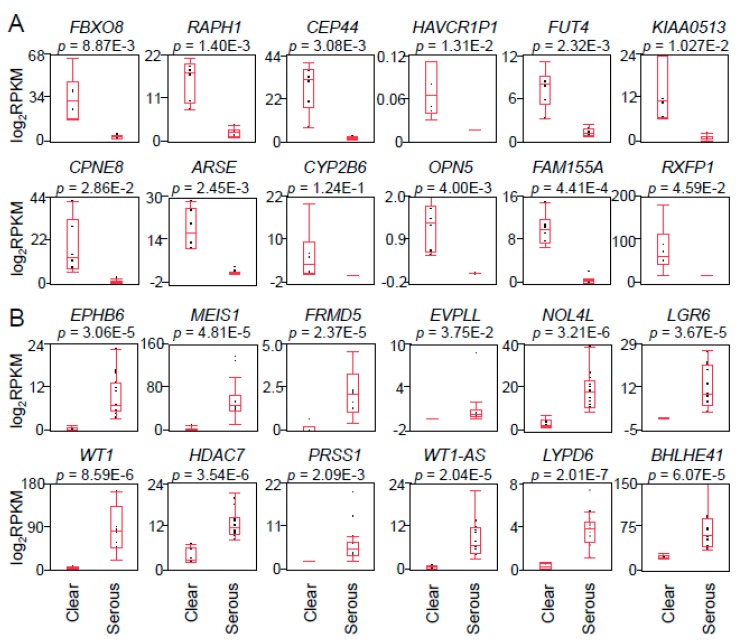
Expression levels of genes contributing to the first principal component of PCA in CCC and HGSC. (**A**) Expression levels (log2RPKM) of the top 12 genes contributing to PC1 negative in Figure 1E were collected from RNA-sequencing data. The box plot was created by the statistical analysis software JMP. Higher expression levels were shown in CCC compared with HGSC. (**B**) Expression levels (log2RPKM) of the top 12 genes contributing to PC1 positive in Figure 1E were collected from RNA-sequencing data. Higher expression levels were shown in HGSC compared with CCC. Results are shown as means ± SD. Statistical analysis was performed using Student’s *t* test followed by FDR (5%) analysis.

**Figure 3:**
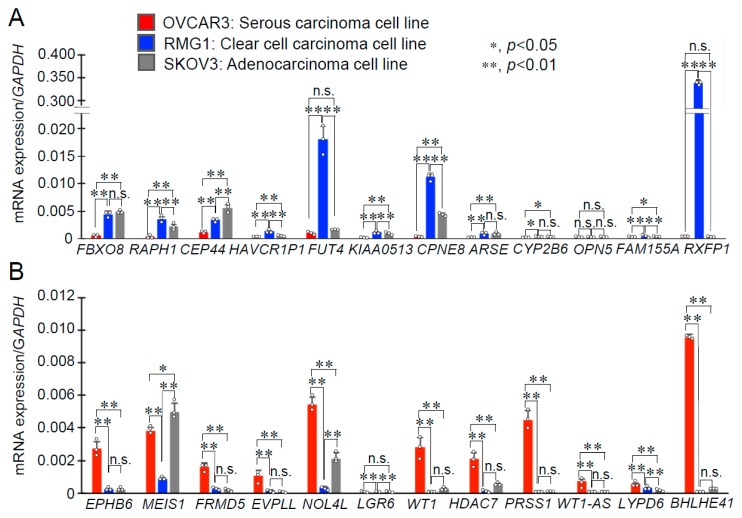
mRNA expression of *CPNE8* and *BHLHE41* in ovarian cancer cell lines. (**A**) The expression levels of the top 12 genes that separated CCC from HGSC (PC1 negativity in Figure 1E) were examined in HGSC-derived OVCAR3, CCC-derived RMG1, and adenocarcinoma-derived SKOV3 cells by qRT-PCR. (**B**) The expression levels of the top 12 genes that separated HGSC from CCC (PC1 positivity in Figure 1E) were examined in OVCAR3, RMG1, and SKOV3 cells by qRT-PCR. Relative mRNA levels are expressed as means ± SD (*n* = 3), by normalizing to *GAPDH* level. Row data are plotted with circles; *, *p* < 0.05; **, *p* < 0.01, using one-way ANOVA with Tukey’s HSD test; n.s., not significant.

To elucidate molecular targets that will be clinically useful for CCC and HGSC, we focused on the genes which contributed to PC1 in the comparison between CCC and HGSC (Figure 1E). The 12 highest positive genes and the 12 lowest negative genes in PC1 are shown in Figure 2A,B, respectively. Box-and-whisker plots were generated based on the RPKM or mRNA expression levels of the 24 genes (Figure 2A,B). As shown in Figure 2, the genes with PC1 negativity (except CYP2B6) were preferentially expressed in CCC versus HGSC (*p* < 0.05; false discovery rate (FDR) 5%). On the contrary, the genes with PC1 positivity were preferentially expressed in HGSC versus CCC (*p* < 0.05; FDR 5%). We next quantified the expression levels of these genes in prototypic ovarian cancer cell lines including RMG1, OVCAR3, and SKOV3, which were primarily established from CCC, HGSC, and adenocarcinoma, respectively (Figure 3). Among them, fucosyltransferase 4 (*FUT4*), *CPNE8*, and relaxin family peptide receptor 1 (*RXFP1*) were the top three most highly expressed genes in RMG1 cells *versus* OVCAR3 cells (Figure 3A), thus we defined them as potential CCC-related markers. Vice versa, nucleolar protein 4 like (*NOL4L*), serine protease 1 (*PRSS1*), and *BHLHE41* were the top three most highly expressed genes in OVCAR3 cells versus RMG1 cells (Figure 3B), and we defined them as potential HGSC-related markers. Adenocarcinoma-derived SKOV3 cells partially expressed both the CCC-related marker *CPNE8* and the HGSC-related marker *NOL4L* (Figure 3).

### 2.2. Functional Analysis of Potential Oncogenes in CCC and HGSC

**Figure 4:**
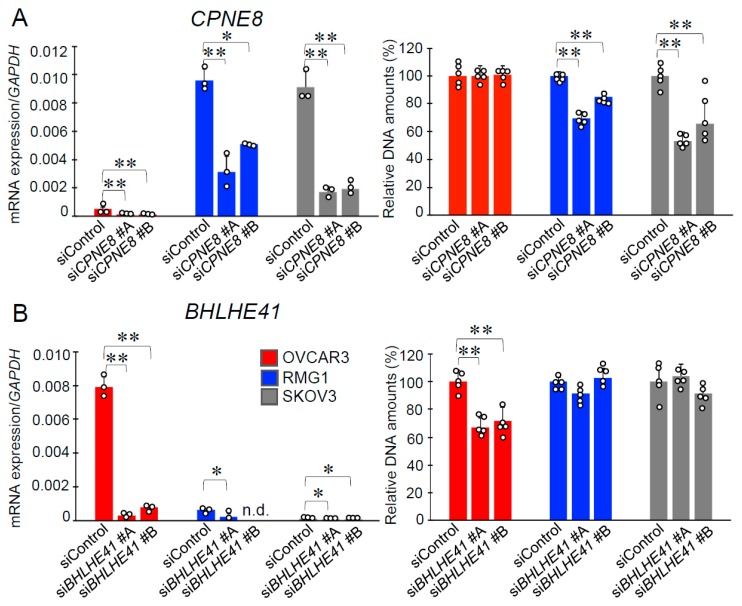
Growth inhibitory effects of siRNAs targeting *CPNE8* and *BHLHE41* in ovarian cancer cells. (**A**) The knockdown efficiency of *CPNE8*-specific siRNAs (si*CPNE8* #A and #B) was examined in OVCAR3, RMG1 and, SKOV3 cells (*n* = 3, left panel). A DNA assay was performed to assess cell growth 4 days after si*CPNE8* #A, #B, or control siRNA (siControl) transfection (*n* = 5, right panel). (**B**) The knockdown efficiency of *BHLHE41*-specific siRNAs (si*BHLHE41* #A and #B) (*n* = 3, left panel) and its effect on cell growth (*n* = 5, right panel) were examined as in (A). Row data are indicated by circles. Results are shown as means ± SD; *, *p* < 0.05; **, *p* < 0.01, using one-way ANOVA with Tukey’s HSD test; n.d., not detected.

Among 24 representative genes that were preferentially overexpressed in either CCC or HGSC (Figure 2), small interfering RNAs (siRNAs) targeting *CPNE8*, *RAPH1*, *CEP44*, *BHLHE41*, *MEIS1*, and *FRMD5* exhibited efficient knockdown in both RMG1 and OVCAR3 cells. We next performed cell proliferation assays using these siRNAs. We showed that siRNAs targeting *CPNE8* (si*CPNE8*) and *BHLHE41* (si*BHLHE41*) could impair cell growth in a histotype-specific manner (Figure 4). Namely, while si*CPNE8* #A and #B significantly downregulated *CPNE8* expression in the three cell lines, only the growth of RMG1 and SKOV3 cells could be repressed by these siRNAs (Figure 4A). In regard to si*BHLHE41* #A and #B that significantly repressed *BHLHE41* expression in the three cell lines, these siRNAs could substantially inhibit the growth of OVCAR3 cells only (Figure 4B). *CPNE8* and *BHLHE41* are also highly expressed in CCCs and HGSCs, respectively, according to three datasets of the online database Oncomine (Supplementary Figure S2).

### 2.3. Pathway Genes Regulated by CPNE8 and BHLHE41

**Figure 5:**
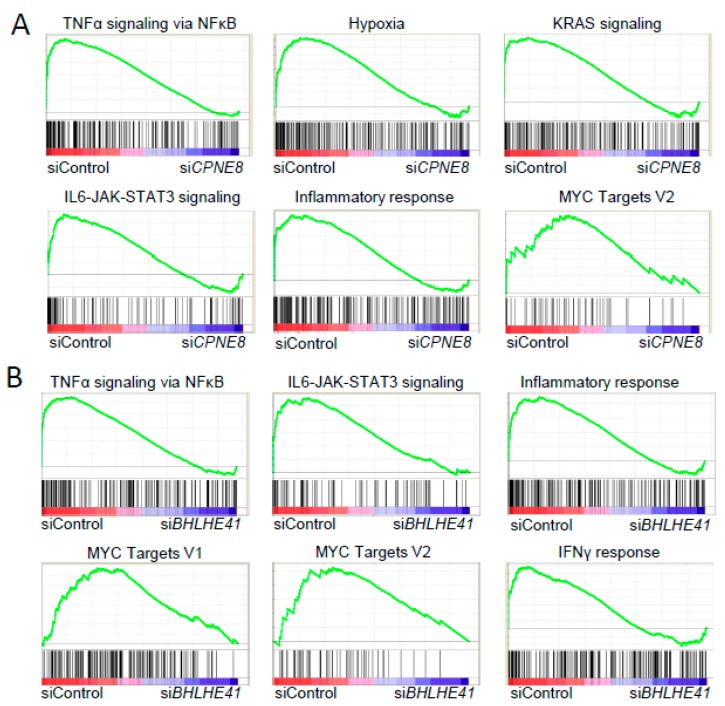
Enriched pathways observed with siRNAs targeting *CPNE8* and *BHLHE41*. (**A**) Gene Set Enrichment Analysis (GSEA) enrichment plot of microarray analysis of RMG1 cells in siControl versus *CPNE8*-specific siRNAs. (**B**) GSEA enrichment plot of microarray analysis of OVCAR3 cells in siControl versus *BHLHE41*-specific siRNAs. The X and Y axes represent “rank in ordered dataset” and “enrichment score”, respectively.

To address the roles of *CPNE8* in the pathophysiology of CCC, we next examined the effects of siRNAs targeting *CPNE8* on the alteration of gene expression in RMG1 cells. Gene set enrichment analysis (GSEA) based on microarray data of RMG1 cells transfected with either *CPNE8* siRNAs or control siRNA dissected functional pathways that were particularly repressed by *CPNE8* knockdown, including NF-κB signaling, hypoxia, KRAS signaling, IL6–JAK–STAT signaling, inflammatory response, and MYC targets (Figure 5A). In addition, functional annotation analysis using DAVID Bioinformatics Tools indicated that the genes upregulated or downregulated commonly by both si*CPNE8* #A and #B treatments defined potential *CPNE8*-regulated pathways in CCC (Supplementary Figure S3A). Notably, functional pathways associated with the response to wounding and cell proliferation were featured by the knockdown study of *CPNE8*.

Similarly, we performed microarray analysis of OVCAR3 cells transfected with siRNAs targeting *BHLHE41* and siControl to address the roles of *BHLHE41* in the pathophysiology of HGSC. GSEA dissected functional pathways that were particularly repressed by *BHLHE41* siRNAs, including NF-κB signaling, IL6–JAK–STAT signaling, inflammatory response, Myc targets, and IFNγ response (Figure 5B). In addition, functional annotation analysis using DAVID Bioinformatics Tools indicated that the genes upregulated or downregulated commonly by both si*BHLHE41* #A and #B treatments depicted potential *BHLHE41*-regulated pathways in HGSC (Supplementary Figure S3B). In particular, inflammatory response and cytokine-related pathways were enriched in relation to genes downregulated by *BHLHE41* siRNAs.

## 3. Discussion

**Figure 6:**
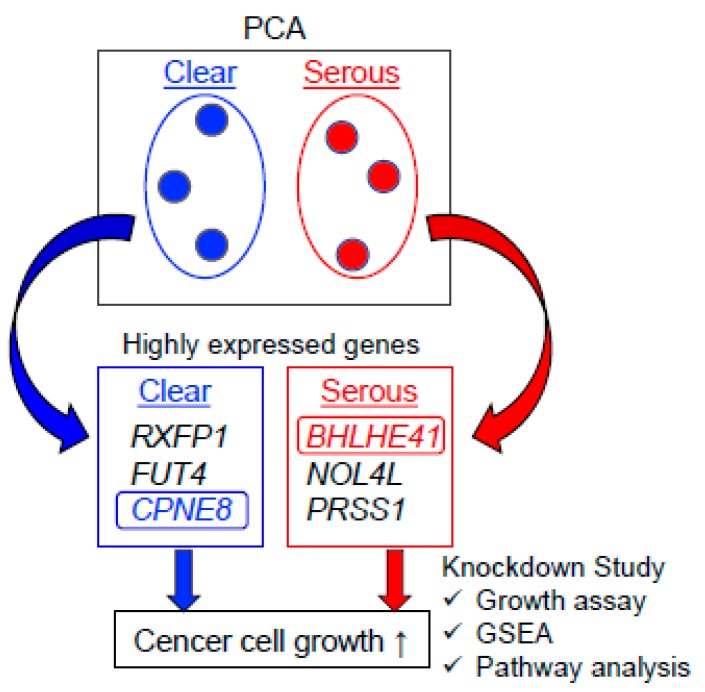
Schema of the present study. PCA based on mRNA levels analyzed by RNA sequencing was conducted in CCC and HGSC groups. The top three most highly expressed genes were chosen from the genes contributing to the first principal component of PCA in CCC and HGSC. Knockdown experiments for the selected genes showed that *CPNE8* and *BHLHE41* promoted the proliferation of CCC- and HGSC-derived cells, respectively. Functional enrichment and pathway analyses also showed that these two genes were related to cancer cell growth signaling.

We performed PCA based on the expression levels of RefSeq genes in clinical ovarian specimens, which were quantified as log2RPKM values by RNA sequencing, as summarized in Figure 6. The PCA-based separation of genes specifically enriched in CCC and HGSC and subsequent functional annotation analysis provided useful information for functional pathways. We assume that the gene expression profiles unique to CCC and HGSC will help to understand the histotype-specific biology of ovarian cancer and to further develop new therapeutic options.

Similar to our study, transcriptomic analysis revealed the expression profiles of ovarian cancer subtypes. Fridley et al. performed RNA-seq to identify differentially expressed genes in HGSC, CCC, and endometrioid carcinoma (EC) [23]. In this report, increased expression levels of *FBXO8*, *RAPH1*, *CEP44*, *KIAA0513*, *FAM155A*, and *RXFP1* were found in CCC compared to HGSC, similar to our study. Zorn et al. investigated the transcriptomic expression profiles of CCC, papillary serous carcinoma, and EC using microarray analysis [24]. Notably, CCC was shown to express high levels of *ARSE* and low levels of *MEIS1*, *HDAC7*, and *PRSS1* compared to other subtypes. In addition, Schaner et al. indicated that *WT1* mRNA level was lower in clear cell carcinomas than in other ovarian epithelial cancers including serous papillary carcinoma, endometrioid carcinoma, undifferentiated carcinoma, and adenocarcinoma [25]. Immunological analysis also revealed histotype-specific markers in ovarian cancer [26]. CCCs are typically negative for WT1 and ESR1 in immunohistochemistry, whereas HGSCs are positive [26]. In the present study, the RPKM values of *WT1* and *ESR1* in CCC (3.6 ± 3.0 and 0.36 ± 0.25, respectively) were small compared to those in HGSC (92.8 ± 51.0 and 18.7 ± 9.6, respectively). CCC were also reported to be positive for HNB1 by immunohistochemistry [18,27]. In the present study, high expression levels of *HNB1* were detected in CCC (RPKM value: 61.7 ± 26.4) but not in HGSC (RPKM value: 0.48 ± 1.15). These similar gene expression patterns support our results. Moreover, the distinct regulation patterns of other genes may be caused by variables including racial differences.

Knockdown experiments showed that *CPNE8* and *BHLHE41*—the former was CCC-predominant, and the latter was HGSC-predominant in PC1 by PCA—regulate the growth of ovarian cancer cells in a histotype-preferential manner. The expression levels of these genes were validated in the Oncomine database.

*CPNE8*, which encodes Copine 8 that belongs to the Copine family, was preferentially expressed in ovarian CCC compared to HGSC. Notably, *CPNE8* knockdown substantially repressed the growth of CCC-derived RMG1 cells but not of HGSC-derived OVCAR3 cells. GSEA showed that *CPNE8* expression positively correlated with the activation of several signaling pathways including NF-kB, STAT, and hypoxic or inflammatory conditions. The Copine family members are considered to be calcium-dependent, phospholipid-binding proteins that may play roles in membrane trafficking [28,29,30]. Several studies have implicated that the Copine family contributes to oncogenesis. In breast cancer, the promoter of the *CPNE8* gene is more frequently methylated in hormone receptor-positive cancer compared with in hormone receptor-negative cancer, thus the difference of the methylation status may partly explain the correlation between *CPNE8* expression and aggressiveness of breast cancer [31]. Since the expression of estrogen and progesterone receptors is almost negative in CCC [26], the gene expression profile of CCC might exhibit a similarity with that of triple-negative breast cancer. Moreover, in basal-like breast cancer cells, *CPNE8* is upregulated by the transcription factor ΔNp63α that stimulates cell migration [32] and is also a target of the tumor suppressive miRNA miR-375 [33]. Thus, CCC may also possess similar characteristics to basal-like breast cancer. In regard to other members of the Copine family, Copine 1 abolishes NF-κB transcription by endoprotease processing of the N-terminus of p65 in human prostate cancer cells [34]. High expression of Copine 1 is also associated with cell growth and metastasis in lung adenocarcinoma [35]. Copine 3 is found as a novel player in the regulation of ErbB2-dependent cancer cell motility in breast cancer T47D cells [36]. This report also showed that Copine 3 expression is increased significantly in metastatic prostate cancer compared with normal prostate and nonmetastatic tumors and in ovarian endometrioid carcinomas compared with normal ovarian tissues. These findings suggest that the Copine family including *CPNE8* would contribute to tumorigenesis, as exemplified by ovarian CCC.

*FUT4* (fucosyltransferase 4) is reported as a biomarker for the diagnosis of breast cancer [37]. This gene is also related to epithelial–mesenchymal transition and invasion of lung cancer [38]. *RXFP1* (relaxin family peptide receptor 1) is known to have an important function in tumor growth and tissue invasion and is related to glioblastoma [39]. Additionally, relaxin is reported to promote prostate cancer progression [40]. In our study, *FUT4* and *RXFP1* were highly expressed in CCC and CCC-derived RMG1 cells. Further experiments will be required to define their functions in ovarian CCC.

*BHLHE41* (basic helix-loop-helix family member e41) is also known as *DEC2* (differentiated embryonic chondrocyte gene 2)/*SHARP1* (split and hairy-related protein 1), encodes a basic helix-loop-helix transcription factor, and is associated with the regulation of apoptosis and cell proliferation in various cancers. In the present study, GSEA revealed that *BHLHE41* knockdown modulated the expression of genes enriched in the NF-kB and MYC pathways and inflammatory conditions. *BHLHE41* was reported to have anti-apoptotic effects on paclitaxel-induced apoptosis in human breast cancer MCF-7 cells [41]. Moreover, *BHLHE41* overexpression increased the proliferation of MCF-7 cells with the upregulation of Myc expression [42]. On the other hand, some studies also reported tumor suppressive functions of *BHLHE41*. For example, *BHLHE41* suppressed tumor proliferation and metastasis by regulating ERK/NF-κB pathway in gastric cancer [43]. Another report indicated that overexpression of *BHLHE41* in endometrial cancer Ishikawa and HEC-1B cells inhibited cell migration, invasion, and metastasis by attenuating *NOTCH1* signaling [44]. Therefore, the contribution of *BHLHE41* to tumor progression remains controversial and possibly depends on the tumor origin or stage. It is reported that aberrant Myc signaling is required for carcinogenesis of fallopian tube secretory epithelial cells, which are considered as an origin of ovarian HGSC [45,46]. These findings imply that *BHLHE41* plays a promotive role in the development of ovarian HGSC, similar to breast cancer.

*NOL4L* (nucleolar protein 4 like) is also known as *C20orf112*. This gene is reported to be one of the fusion partners of *RUNX1* (runt-related transcription factor 1) in acute myeloid leukemia and of *PAX5* (paired box 5) in acute lymphoblastic leukemia; however, its precise role is still to be clarified [47,48]. *PRSS1* (serine protease 1) is a gene whose germline variants are implicated in hereditary pancreatitis and an increased risk of pancreatic ductal adenocarcinoma [49]. It is also related to pediatric cancer, such as brain tumors or Wilms tumors [50]. In the present study, both of these genes were highly expressed in HGSC and HGSC-derived OVCAR3 cells, although siRNAs targeting either of these genes did not modulate the growth of OVCAR3 cells apparently (data not shown). [51] Further study will be required to clarify their functions in ovarian HGSC.

Interestingly, adenocarcinoma-derived SKOV3 cells partially expressed both CCC- and HGSC-related markers. Although it has been used widely as an ovarian cancer model, SKOV3 cell line is now considered unsuitable as an HGSC model [52,53,54]. On the other hand, it was reported that intraperitoneal injection of SKOV3 cells into nude mice generated CCC tumors loosely adherent to the fat tissue [55]. Therefore, SKOV3 cells partially share biomarkers associated with both CCC and HGSC, suggesting undifferentiated characteristics.

As mentioned above, CCC represent only 10% of ovarian cancer in Caucasians, hence, few numbers of clinical datasets exist in public databases. Therefore, fewer molecular targets have been identified for ovarian CCC compared with HGSC. We performed a systematic gene identification for these two histotypes of Japanese ovarian cancer and showed for the first time that *CPNE8* plays oncogenic roles in ovarian CCC. This gene could be used as a biomarker and therapeutic target for CCC. We also found sets of genes that were defined as contributing genes for CCC in PC1. Additional functional analysis for these genes is necessary to reveal new targets and determine precise molecular mechanisms of ovarian CCC.

As a limitation of this study, we did not perform particular enrichment procedures such as microdissection to collect cancer tissues with increased tumor purity from ovary tumor samples. As shown in a study of tumor purity estimation in a The Cancer Genome Atlas (TCGA) pan-cancer analysis, tumor purity would have an impact on the results of omics analyses [56]. Tumor purity differences resulting from sampling variation may exceed intrinsic individual differences, thus tumor purity should be considered in future cancer-related analyses of genomic datasets. The sample size was also a limitation in this study. A future comparison study among histotypes using a larger sample size will define the precise histotype-specific regulation and function of genes in ovarian cancer. In this study, we evaluated the relevance of the genes identified from clinical sequence data in ovarian cancer cell lines. Further in vitro experiments evaluating migration and invasion abilities and therapy response would support the significance of the clinical analysis.

Taken together, the systemic identification of differentially expressed genes in CCC and HGSC will enlighten us on the differences regarding the predominant pathways in these cancers. In the present study, we systemically identified potential ovarian cancer-related genes differentially expressed in CCC and HGSC based on the PCA of gene expression levels determined by RNA sequencing. The present results will be applied to the development of potential diagnostic and therapeutic options for the disease.

## 4. Materials and Methods

### 4.1. Clinical Specimens and Cell Lines

Experiments using patient data and specimens were approved by the Saitama Medical University International Medical Center Institutional Review Board (#13-165, 7 Feb 2014). Tumor or normal specimens were available from 27 patients who underwent surgery for primary ovarian tumor with their informed consent in a previous study (#12-096, 5 Sep 2012): 6 clear cell carcinomas (CCCs), 15 high-grade serous carcinomas (HGSCs), and 6 normal tissues. All tumor specimens were obtained at surgery from newly diagnosed primary tumors without neoadjuvant chemotherapy and used for RNA extraction without selecting a particular region of the tumors. These patients included both premenopausal and menopausal women (Supplementary Table S1). Among six normal tissues, five tissues were obtained from a scrape biopsy of the contralateral unaffected ovary in patients with unilateral ovary tumor. One normal tissue was derived from the ipsilateral oviduct of a benign ovarian tumor (mucinous cyst adenoma). All of the normal samples did not include malignant lesions. The human ovarian cancer cell lines OVCAR3 and SKOV3 were grown in DMEM with 10% fetal bovine serum (FBS) and 1% penicillin/streptomycin at 37 °C under 5% CO_2_. Another human ovarian cancer cell line, RMG1, was grown in RPMI 1640 with 10% FBS and 1% penicillin/streptomycin at 37 °C under 5% CO_2_. All cell lines were authenticated by short-tandem-repeat (STR) analysis (BEX, Tokyo, Japan).

### 4.2. RNA Sequencing

RNA was extracted from fresh frozen tissues using NucleoSpin RNA (Takara, Japan). All the RNA samples were extracted with the same batch and validated as optimal samples with an RNA integrity number (RIN) value > 8.0. An RNA library was prepared by using the SureSelect Strand Specific RNA Library Prep Kit (Agilent, Palo Alto, CA, USA), and 100 bp paired-end RNA-sequencing was performed via HiSeq2500^®^ (Illumina, San Diego, CA, USA). Sequence analysis was performed in two batches, and the %PF (% clusters passing filter) values for sequence run were ranged in 94.8–95.3 for one batch (including four serous cancer samples) and 86.5–94.5 for the other batch (including the rest of the samples). As a quality control check, we performed FastQC and confirmed that all the samples exhibited good sequence quality, with mean-per-base sequence quality >26. After deleting ribosomal RNA and adapter sequence from the merged FASTQ data, sequence tags were aligned to the human genome assembly hg19, using TopHat based on the Bowtie algorithm, and the expression values were quantified as read per kilobase of transcript length per million mapped reads (RPKM) based on the RefSeq gene model. The RPKM data are provided in Supplementary Data S1.

### 4.3. siRNA and Transfection

Small interfering RNAs (siRNAs) against *CPNE8* and *BHLHE41* were synthesized or purchased commercially (Sigma-Aldrich, St Louis, MO, USA) as follows: si*CPNE8* #A, 5′-CUGAGCAGUUUCUCUCCUAUA-3′ (sense) and 5′-UAGGAGAGAAACUGCUCAGGG-3′ (antisense); si*CPNE8* #B, 5′-AUCAGAUUUAGAAAAUGUGUC-3′ (sense) and 5′-CACAUUUUCUAAAUCUGAUCC-3′ (antisense); si*BHLHE41* #A, 5′-CACGUUGCAACCUAUUCUGAA-3′ (sense) and 5′-CAGAAUAGGUUGCAACGUGAG-3′ (antisense); si*BHLHE41* #B, 5′-GCCAUCGUCAGAACUAAGUCA-3′ (sense) and 5′-ACUUAGUUCUGACGAUGGCCC-3′ (antisense). Control siRNA was purchased commercially (RNAi Inc., Japan) as follows: siControl, 5′-GUACCGCACGUCAUUCGUAUC-3′ (sense) and 5′-GAUACGAAUGACGUGCGGUAC-3′ (antisense). These siRNAs were transfected into OVCAR3, RMG1, and SKOV3 cells using Lipofectamine RNAiMax (Invitrogen, Carlsbad, CA, USA) in the absence of penicillin/streptomycin according to the manufacturer’s instruction when the cells reached 40–60% confluence. After 48 h, the cells were harvested to analyze the knockdown efficiency using qRT-PCR.

### 4.4. RNA Isolation and Quantitative RT-PCR Analysis

Total RNA was isolated from OVCAR3, RMG1, and SKOV3 cells using ISOGEN (Nippongene, Japan). One microgram of total RNA was reverse-transcribed to cDNA by SuperScript III (Invitrogen, Carlsbad, CA, USA) in a final volume of 20 μL. Then, 0.067 μL of cDNA was used to perform quantitative PCR reactions with KAPA SYBR^®^ FAST ABI Prism^®^ (Kapa Biosystems, Foster City, CA, USA) on Step One Plus (Applied Biosystems, Foster City, CA, USA). *GAPDH* was used as the normalization control. The primers are shown in Supplementary Table S2.

### 4.5. Cell Proliferation Assay

Cell proliferation was estimated by a DNA assay. In brief, OVCAR3, RMG1, and SKOV3 cells plated onto 96-well plates were transfected with siRNAs and then incubated for 4 days. A DNA assay was performed using the fluorochrome Hoechst 33258 to quantify cellular DNA content in 96-well tissue cultures plates [57]. Five replicate wells (*n* = 5) were used for the cell proliferation assay. Fluorescence intensity was measured using an ARVO microplate reader (PerkinElmer, Foster City, CA, USA).

### 4.6. Microarray Analysis

RMG1 cells were transfected with si*CPNE8* #A and #B and siControl. OVCAR3 cells were transfected with si*BHLHE41* #A and #B and siControl. Total RNA was isolated from these cells using ISOGEN and subjected to microarray analysis using Affymetrix GeneChip (Clariom S Array). The microarray data were deposited in Gene Expression Omnibus under accession number GSE125542. A global analysis of gene expression, differentially expressed genes in pathways, and clusters of functionally related genes was performed using the DAVID Bioinformatics Resources 6.7 (https://david-d.ncifcrf.gov/summary.jsp) and Gene Set Enrichment Analysis (GSEA) (http://software.broadinstitute.org/gsea/index.jsp).

### 4.7. Principal Component Analysis

PCA was performed with R-package 3.2.2 (available from www.Bioconductor.org) using RPKM values of RefSeq genes. Correlating ratios of principal component 1 and 2 (PC1 and PC2) were calculated. Functional annotation and pathway enrichment analysis were performed with DAVID Bioinformatics Resources 6.7 (available from https://david-d.ncifcrf.gov/) using about 500–1000 genes, which showed >0.8 or <-0.8 of correlation coefficient value to PC1.

### 4.8. Statistical Analysis

For comparison of the RPKM values (or expression levels) between CCC and HGSC shown in Figure 2, unpaired Student’s *t*-test was performed, and the genes satisfying the criterion of false discovery rate (FDR) 5% were considered to be significant. One-way analysis of variance (ANOVA) was used with Tukey’s honestly significant difference (HSD) test for multiple comparisons in experiments using three cell lines (Figure 3 and Figure 4).

### 4.9. Validation of Gene Expression Levels

Gene expression levels were validated on the basis of the online database Oncomine (https://www.oncomine.org/).

### 4.10. Data Availability Statement

The microarray data have been deposited in Gene Expression Omnibus under accession number GSE125542.

## Supplementary Materials

Supplementary materials can be found at https://www.mdpi.com/1422-0067/20/18/4330/s1.
