# Supplementary material for: Systematic Identification of Characteristic Genes of Ovarian Clear Cell Carcinoma Compared with High-Grade Serous Carcinoma Based on RNA-Sequencing
Source: Int J Mol Sci. 2019 Sep 4;20(18):4330. doi: 10.3390/ijms20184330 (PMC6770582; doi:10.3390/ijms20184330)
Supplement: Supplementary file 1 [file ijms-20-04330-s001.zip › Supplementary Information.pdf]

Supporting Information

Systematic identification of characteristic genes of ovarian clear cell carcinoma compared with high-grade serous carcinoma based on RNA-sequencing

Saya Nagasawa, Kazuhiro Ikeda, Kuniko Horie-Inoue, Sho Sato, Atsuo Itakura, Satoru Takeda, Kosei Hasegawa, Satoshi Inoue

Supplementary Table S1. Clinicopathological characteristics of 21 ovarian cancers and 6 normal tissues

Supplementary Table S2. Sequence of primers for qRT-PCR

Supplementary Table S3. Top 10 pathways enriched in clear cell carcinoma *versus* normal tissues

Supplementary Table S4. Top 10 pathways enriched in normal tissues *versus* clear cell carcinoma

Supplementary Table S5. Top 10 pathways enriched in high-grade serous carcinoma *versus* normal tissues

Supplementary Table S6. Top 10 pathways enriched in normal tissues *versus* high-grade serous carcinoma

Supplementary Table S7. Top 10 pathways enriched in clear cell carcinoma versus high-grade serous carcinoma

Supplementary Table S8. Top 10 pathways enriched in high-grade serous carcinoma versus clear cell carcinoma

Supplementary Figure S1. Pathway enrichment analysis using PC1-contributing genes in PCA.

Supplementary Figure S2. Gene expression levels of *CPNE8* and *BHLHE41* based on Oncomine.

Supplementary Figure S3. Up- and down-regulated pathway genes by siRNAs targeting *CPNE8* and *BHLHE41*.

Supplementary Table S1. Clinicopathological characteristics of 21 ovarian cancers and 6 normal tissues

Characteristics	Ovarian cancer subtype or normal tissue		
	Clear cell	High-grade serous	Normal tissue*
Total cases	6	15	6
Patient age			
Average	57.5	60.3	53.7
Median	59.5	61.0	53
Range	50-63	40-80	41-66
Menopause			
Premenopause	1	3	3
Menopause	5	12	3
FIGO Stage			
I, II	3	0	
III, IV	3	15	

*Among 6 normal tissues, 5 tissues were obtained from scrape biopsy of the contralateral unaffected ovary in patients with unilateral ovary tumor and one normal tissue was derived from ipsilateral oviduct of benign ovarian tumor (mucinous cyst adenoma).

Supplementary Table S2. Sequence of primers for qRT-PCR

Target Gene	Forward Primer	Reverse Primer
EPHB6	ATCCAGCTCCTTCAGCAACAC	GAGTCACGGGTATCGTCATTCTC
MEIS1	GCACACTGAGTCTTAGCGTTTCTG	CAACTGGGCTTGGCGTTATT
FRMD5	GAGCTGTTCACATCTCCATCATG	GAACGCACTGGTGTGGAATG
EVPLL	GAGGACTACAGCCGGATTCTGT	CCAGAAATCCCCTTCATGCA
NOL4L	GGCAAGAGCGTAGGTTCAATGT	AGGCCCAATGGACTTTAACAAC
LGR6	TGTGGTAGGTGCGATTGCA	GGGCATCGACTGAGGCTAGA
WT1	ACAGGGTACGAGAGCGATAACC	CCGTGCGTGTGTATTCTGTATTG
HDAC7	CAAGAGCAAGCGAAGTGCTGTA	TTCAGAATCACCTCCGCTAGCT
PRSS1	TTCTGTGTGGGCTTCCTTGAG	CACAGGGCCACCAGAATCA
WT1-AS	GAACCACCGATTGGCAAAGA	CCTCAGTTTCCTTCTCTGTAAAACG
LYPD6	TCTCAGTCACCAAACGCTGTGT	GAGTCTCTGCAGCCAGTGGATA
BHLHE41	TAACCGCCTTAACCGAGCAA	TCAGAGATCGCTCCCCATTC
FBXO8	CCATCTTGTCCTACCTGAATGCA	TCGCAAGGTCCTGCCAAA
RAPH1	AGGGCAGGCTGTGTCAAAGT	GTTTGAATGGTGACAGGATGCA
CEP44	GGTGCTCCGCTTGCTAAATT	CTGCTGGGTCTCCCTTTATCAA
HAVCR1P1	CTGGAATAGAGGCGCACGTT	GGGTTCCATTGGTCCAGACA
FUT4	TGAGACGGAGTCTTGCACTGTAG	GAGGTTGCAGTGAGCCAAGAT
KIAA0513	TCTTAGGATGCAGCCGTCTCA	GACGCAGCTGGGAAAAAGTG
CPNE8	CAATATGGACAGCCGCTACAAC	GCGCTCAGCTGGTTCAAGTC
ARSE	AGGGAAGCTCACACACCTGATAC	CGAAAGGGCTGACCAGATGA
CYP2B6	ATGGAAACCGCTGGAAGGT	GCTTTCCCATCCCGAAGTC
OPN5	GTGAAAGCTGGCCTGAAAAGC	GAGTGTAGCTGCTCCAGTCAGAGA
FAM155A	GGCTCAGAAATGGCGACTGT	GTGATCAGAGAGCAGGACTGTGA
RXFP1	GCCTCAGCTCCTGCACTGTAA	TGTTGTCTCCACAGTTGTCCTCAT

Supplementary Table S3. Top 10 pathways enriched in clear cell carcinoma *versus* normal tissues

GO Term	Count	*P* value	Genes
Cell cycle phase	86	8.67E-33	*KIF23, E2F1, KIFC1, KIF22, XRCC2, PRC1, KNTC1, PKMYT1, TTK, AURKA, AURKB, PTTG1, CD2AP, GTSE1, FAM83D, CUL2, ACVR1B, KIF2C, CCNE1, CDCA8, CDKN2A, CDCA2, TUBG1, FANCA, CCNA2, CDCA5, ASPM, CDCA3, CDC6, CDK1, KIF11, SGOL1, KIF15, CCNF, TPX2, MND1, ESPL1, PBK, TACC3, NCAPD3, RAD51, MAD2L1, TIMELESS, SPAG5, FANCD2, ZWINT, BUB1B, USH1C, KPNA2, BLM, NEK2, USP9X, ANLN, CHEK1, CEP55, SPC25, NCAPH, NCAPG, NCAPG2, OVOL1, BUB1, SKA3, SKA1, ERCC6L, TRIP13, EXO1, MKI67, KIF18A, NUF2, BRCA2, CENPF, CDC20, CENPE, NDC80, BIRC5, SIRT7, CDKN3, RAD54L, SMC4, CCNB1, CCNB2, PLK1, CKS2, RAD54B, CIT, DNM2*
M phase	76	4.02E-32	*KIF23, KIFC1, KIF22, XRCC2, PRC1, KNTC1, PKMYT1, TTK, AURKA, AURKB, PTTG1, CD2AP, FAM83D, KIF2C, CDCA8, CDCA2, TUBG1, FANCA, CCNA2, CDCA5, ASPM, CDCA3, CDC6, CDK1, KIF11, SGOL1, CCNF, KIF15, TPX2, MND1, ESPL1, PBK, TACC3, NCAPD3, RAD51, MAD2L1, TIMELESS, SPAG5, FANCD2, ZWINT, BUB1B, KPNA2, NEK2, USP9X, ANLN, CHEK1, CEP55, SPC25, NCAPH, NCAPG, NCAPG2, OVOL1, BUB1, SKA3, SKA1, ERCC6L, TRIP13, EXO1, MKI67, KIF18A, NUF2, BRCA2, CENPF, CDC20, BIRC5, NDC80, CENPE, SIRT7, RAD54L, SMC4, CCNB1, CCNB2, PLK1, CKS2, RAD54B, CIT*
Cell cycle	118	9.59E-32	*KIFC1, XRCC2, PRC1, DTYMK, KNTC1, PKMYT1, TTK, AURKA, AURKB, PTTG1, CD2AP, CUL2, CDCA8, CDKN2A, CDCA2, TUBG1, CCNA2, CDCA5, ASPM, CDCA3, SGOL1, MND1, ESPL1, TACC3, NCAPD3, ESCO2, PPP1CA, UHRF1, KRT18, MAD2L1, TIMELESS, SPAG5, PSMA5, ZWINT, PSMA3, BLM, PKHD1, NEK2, CHEK1, ANLN, CALR, LLGL2, SPC25, NCAPG2, PSMB3, OVOL1, SKA3, SKA1, ERCC6L, CKAP2, MKI67, NUF2, BRCA2, CDC20, NDC80, RAD54L, BRCA1, PSMC4, PLK1, RAD54B, CHAF1A, KIF23, E2F1, KIF22, E2F7, GTSE1, CDT1, FAM83D, CCNE1, ACVR1B, KIF2C, FANCI, PSMD6, ARHGAP8, FANCA, PSMD8, CDK1, CDC6, KIF11, CCNF, KIF15, TPX2, PBK, CDK7, MCM2, CDK5, RAD51, FANCD2, USH1C, BUB1B, PSME3, KPNA2, USP9X, FOXM1, CEP55, NCAPH, NCAPG, HJURP, BUB1, TRIP13, EXO1, KIF18A, CENPF, BIRC5, CENPE, SIRT7, RACGAP1, CDKN3, GSG2, SMC4, CCNB1, PSMD14, CCNB2, PSMD12, PTP4A1, CKS2, CIT, DNM2*
Mitotic cell cycle	80	1.02E-31	*KIF23, E2F1, KIFC1, KIF22, PRC1, KNTC1, PKMYT1, TTK, AURKA, AURKB, PTTG1, CD2AP, GTSE1, FAM83D, CUL2, ACVR1B, KIF2C, CCNE1, CDCA8, CDKN2A, CDCA2, PSMD6, CDCA5, CCNA2, ASPM, PSMD8, CDCA3, CDC6, CDK1, KIF11, SGOL1, CCNF,*

			KIF15, TPX2, ESPL1, PBK, NCAPD3, MAD2L1, TIMELESS, SPAG5, PSMA5, ZWINT, PSMA3, BUB1B, USH1C, PSME3, KPNA2, BLM, NEK2, USP9X, ANLN, CHEK1, CEP55, SPC25, NCAPH, NCAPG, NCAPG2, PSMB3, BUB1, SKA3, SKA1, ERCC6L, KIF18A, NUF2, CENPF, CDC20, CENPE, BIRC5, NDC80, SIRT7, CDKN3, SMC4, CCNB1, PSMD14, CCNB2, PSMD12, PSMC4, PLK1, CIT, DNM2
Cell cycle process	98	8.99E-31	*E2F1, KIF23, KIF22, KIFC1, XRCC2, PRC1, KNTC1, TTK, PKMYT1, AURKA, AURKB, PTTG1, CD2AP, GTSE1, FAM83D, KIF2C, ACVR1B, CCNE1, CUL2, CDCA8, CDKN2A, CDCA2, TUBG1, PSMD6, FANCA, CDCA5, CCNA2, ASPM, PSMD8, CDCA3, CDC6, CDK1 KIF11, SGOL1, KIF15, CCNF, TPX2, MND1, ESPL1, PBK, TACC3, NCAPD3, RAD51, MAD2L1, TIMELESS, SPAG5, FANCD2, PSMA5, ZWINT, PSMA3, USH1C, BUB1B, PSME3, KPNA2, BLM, NEK2, USP9X, CHEK1, ANLN, CEP55, CALR, SPC25, NCAPH, NCAPG2, NCAPG, PSMB3, OVOL1, BUB1, SKA3, SKA1, ERCC6L, TRIP13, EXO1, MKI67, KIF18A, NUF2, CENPF, BRCA2, CENPE, BIRC5, NDC80, CDC20, SIRT7, RACGAP1, CDKN3, RAD54L, BRCA1, SMC4, CCNB1, PSMD14, CCNB2, PSMC4, PSMD12, PLK1, CKS2, RAD54B, CIT, DNM2*
M phase of mitotic cell cycle	57	2.64E-26	*KIF23, KIF22, KIFC1, KNTC1, PKMYT1, AURKA, PTTG1, AURKB, CD2AP, FAM83D, KIF2C, CDCA8, CDCA2, CDCA5, CCNA2, ASPM, CDCA3, CDC6, CDK1, KIF11, SGOL1, CCNF, KIF15, TPX2, ESPL1, PBK, NCAPD3, MAD2L1, TIMELESS, SPAG5, ZWINT, BUB1B, NEK2, USP9X, ANLN, CEP55, SPC25, NCAPH, NCAPG, NCAPG2, BUB1, SKA3, SKA1, ERCC6L, KIF18A, NUF2, CENPF, CDC20, BIRC5, NDC80, CENPE, SIRT7, SMC4, CCNB1, CCNB2, PLK1, CIT*
Nuclear division	56	7.49E-26	*KIF23, KIF22, KIFC1, KNTC1, PKMYT1, AURKA, PTTG1, AURKB, CD2AP, FAM83D, KIF2C, CDCA8, CDCA2, CDCA5, CCNA2, ASPM, CDCA3, CDC6, CDK1, KIF11, SGOL1, CCNF, KIF15, TPX2, ESPL1, PBK, NCAPD3, MAD2L1, TIMELESS, SPAG5, ZWINT, BUB1B, NEK2, USP9X, ANLN, CEP55, SPC25, NCAPH, NCAPG, NCAPG2, BUB1, SKA3, SKA1, ERCC6L, KIF18A, NUF2, CENPF, BIRC5, NDC80, CENPE, CDC20, SMC4, CCNB1, CCNB2, PLK1, CIT*
Mitosis	56	7.49E-26	*KIF23, KIF22, KIFC1, KNTC1, PKMYT1, AURKA, PTTG1, AURKB, CD2AP, FAM83D, KIF2C, CDCA8, CDCA2, CDCA5, CCNA2, ASPM, CDCA3, CDC6, CDK1, KIF11, SGOL1, CCNF, KIF15, TPX2, ESPL1, PBK, NCAPD3, MAD2L1, TIMELESS, SPAG5, ZWINT, BUB1B, NEK2, USP9X, ANLN, CEP55, SPC25, NCAPH, NCAPG, NCAPG2, BUB1, SKA3, SKA1, ERCC6L, KIF18A, NUF2, CENPF, BIRC5, NDC80, CENPE, CDC20, SMC4, CCNB1, CCNB2, PLK1, CIT*

Organelle fission	57	8.75E-26	*KIF23, KIF22, KIFC1, KNTC1, PKMYT1, AURKA, PTTG1, AURKB, CD2AP, FAM83D, KIF2C, CDCA8, CDCA2, CDCA5, CCNA2, ASPM, CDCA3, CDC6, CDK1, KIF11, SGOL1, CCNF, KIF15, TPX2, ESPL1, PBK, NCAPD3, MAD2L1, TIMELESS, SPAG5, ZWINT, BUB1B, NEK2, USP9X, ANLN, CEP55, SPC25, NCAPH, NCAPG, NCAPG2, BUB1, SKA3, SKA1, ERCC6L, KIF18A, NUF2, CENPF, CDC20, BIRC5, NDC80, CENPE, SMC4, CCNB1, CCNB2, PLK1, BAX, CIT*
Cell division	61	4.44E-23	*KIF23, KIFC1, PRC1, KNTC1, PTTG1, AURKB, CD2AP, FAM83D, TOP1, CCNE1, CDCA8, CDKN2A, CDCA2, CDCA5, CCNA2, ASPM, CDCA3, CDC6, CDK1, KIF11, SGOL1, CCNF, ESPL1, CDK7, CDK5, NCAPD3, PPP1CA, MAD2L1, TIMELESS, SPAG5, ZWINT, BUB1B, PKHD1, NEK2, USP9X, ANLN, CEP55, LLGL2, SPC25, NCAPH, NCAPG, NCAPG2, BUB1, SKA3, SKA1, ERCC6L, NUF2, CENPF, BRCA2, CDC20, BIRC5, NDC80, CENPE, RACGAP1, SMC4, CCNB1, CCNB2, PLK1, ANXA11, CKS2, CIT*

Supplementary Table S4. Top 10 pathways enriched in normal tissues *versus* clear cell carcinoma

GO Term	Count	*P* value	*Genes*
Transcription	181	4.42E-16	*MEF2C, THRA, TCEAL6, ZNF781, BBX, APOBEC3G, TCEAL7, TCEAL2, TCEAL1, TCEAL4, FOXO6, ZXDA, GTF2IRD2B, TCEAL3, HOXC6, PGR, ZFP92, EPC2, ZNF181, CRY2, CGGBP1, GATA6, ZFP90, PSIP1, ZNF442, ZNF397, ZNF395, RARA, ATOH8, SAP30L, FOXO3B, ZNF575, TWIST2, GABPB2, ZNF43, ZNF641, ZNF594, RCOR3, RXRA, ZHX1, MECP2, GTF2IRD2, HMG20A, ARID1B, TRERF1, PROX1, FOXN3, PRDM8, PTRF, ZNF197, PRDM5, ZNF383, PRDM2, TGFB1I1, ZIM2, CRTC3, ZNF519, TSHZ3, EID1, TSHZ2, ZNF430, ZNF132, TADA3, ZNF514, WT1, ZNF512, NR2C2, ARNT, HTATSF1, TFDP2, TEF, ZNF425, ZNF599, MAML3, TCF4, ASF1A, ZNF70, ASXL3, ZNF529, TAF3, SMAD9, KLF12, SMAD5, TAF7, ZNF621, ZNF521, KLF15, MED13L, ZNF835, FOXP1, SALL2, CSRNP3, PHF1, DMTF1, EBF1, ATF7, ZIK1, KLF2, POU6F1, ZNF83, ZBTB33, BACH2, ELF2, ZNF532, EID2B, ZEB2, GLI2, GLI3, KCNIP3, MAX, ZNF738, HSF2, ANG, CCDC101, ZNF540, ZNF496, ZNF493, NR2F1, ZBTB22, SOX10, AR, ELP2, ZNF280D, FOXJ2, POLR1E, SPEN, ZFP28, ZNF333, ZNF688, NRIP2, PURA, ZMIZ1, ASH1L, ZNF711, ZFPM2, JMJD1C, ZNF33B, ZNF483, SUPT3H, ZNF275, NDN, SCML1, NFIX, ZBTB16, PRDM16, CALCOCO1, HIC1, ZNF660, NPAS3, TSPYL2, NR1D1, HAND2, NR1D2, ZNF708, ZNF286A, PER3, BAZ2B, CHD5, MAF, KAT2B, TBX3, AFF3, ZNF25, SNAI2, ZNF358, ZNF662, ATXN3, PHF19, BNC2, ZNF862, SP4, PHF21A, MAMLD1, DENND4A, ZBTB2, PBX1, PAPOLG, NFIC, APBB1, NFIA, NFIB*
Regulation of transcription	204	5.25E-14	*MEF2C, ZNF781, BBX, GTF2IRD2B, ZFP92, PGR, EPC2, CRY2, ZFP90, ZNF397, ZNF395, RARA, SAP30L,*

			FOXO3B, TIGD7, TWIST2, ZNF43, ZNF641, RCOR3, RXRA, ZHX1, MECP2, HMG20A, PROX1, PRDM8, PTRF, PRDM5, ZNF383, PRDM2, TGFB1I1, ZIM2, CRTC3, ZNF519, ZNF132, TADA3, AFAP1L2, ZNF514, ZNF512, JRK, HTATSF1, MAML3, TCF4, ZNF70, TCF23, BMP4, ASXL3, ZNF529, SMAD9, KLF12, ZNF621, SMAD5, ZNF521, KLF15, SFMBT2, CSRNP3, DMTF1, ZIK1, KLF2, ENG, ZBTB33, BACH2, ELF2, ZNF532, EID2B, ZEB2, GLI2, GLI3, KCNIP3, PBXIP1, ZNF738, HSF2, ZNF540, ZNF496, ZNF493, CSDC2, NR2F1, ZBTB22, AR, SPEN, ZNF333, ZNF688, ASH1L, ZNF33B, ZNF483, ZNF275, SCML1, ZBTB16, ZNF660, NR1D1, NR1D2, CSDE1, ZNF286A, PER3, BAZ2B, TBX3, ZNF25, AFF3, SNAI2, ZNF358, ZNF662, PKNOX2, PHF19, ZNF862, BNC2, PHF21A, ZBTB2, PBX1, APBB1, THRA, CCDC85B, TCEAL6, TCEAL7, TCEAL2, TCEAL1, TCEAL4, FOXO6, ZXDA, TCEAL3, HOXC6, ZNF181, GATA6, CGGBP1, PSIP1, ZNF442, ATOH8, ZNF575, GABPB2, ZNF594, SATB1, GTF2IRD2, ARID1B, TRERF1, FOXN3, KRBA2, ZNF197, EID1, TSHZ3, TSHZ2, ZNF430, NR2C2, WT1, ARNT, MEIS2, TEF, TFDP2, ZNF425, ZNF599, ASF1A, TAF3, RFX7, TAF7, LMCD1, MED13L, ZNF835, FOXP1, SALL2, PHF1, ATF7, EBF1, ABL1, POU6F1, ZNF83, PDCD4, MAX, CCDC101, SOX15, SOX10, ELP2, FOXJ2, ZNF280D, LDB1, ZFP28, NRIP2, PURA, ZMIZ1, ZNF711, ZFPM2, JMJD1C, MAPRE3, SUPT3H, NDN, NFIX, HCFC2, PRDM16, CALCOCO1, HIC1, TSC22D3, RGMB, TSPYL2, NPAS3, HAND2, ZNF708, CHD5, MAF, KAT2B, ATXN3, SP4, MAMLD1, DENND4A, JAK2, ID3, NFIC, NFIA, NFIB
DNA binding	179	4.65E-11	*MEF2C, THRA, ZNF781, BBX, H1FX, RFXAP, TCEAL1, FOXO6, ZXDA, GTF2IRD2B, HOXC6, PGR, ZFP92, ZNF181, CGGBP1, GATA6, ZFP90, PSIP1, ZNF442, ZNF397, ZNF395, RARA, ATOH8, FOXO3B, ZNF575,*

			TIGD7, TWIST2, ZNF43, SATB1, ZNF641, ZNF594, RCOR3, RXRA, ZHX1, MECP2, GTF2IRD2, HMG20A, ARID1B, TRERF1, PROX1, FOXN3, PRDM8, KRBA2, ZNF197, PRDM5, ZNF383, PRDM2, ZIM2, ZNF519, TSHZ3, TSHZ2, ZNF430, ZNF132, TADA3, CXXC4, ZNF514, WT1, ZNF512, NR2C2, ARNT, JRK, RPS27, MEIS2, TFDP2, TEF, ZNF425, ZNF599, TCF4, ZNF70, ZNF529, TAF3, SMAD9, SETDB2, SMG6, KLF12, RFX7, SMAD5, TAF7, ZNF621, ZNF521, KLF15, ZNF835, FOXP1, ATM, SALL2, CSRNP3, PHF1, DMTF1, EBF1, ATF7, ZIK1, KLF2, ABL1, PRKRIR, NYNRIN, POU6F1, ZNF83, ZBTB33, BACH2, ELF2, ZNF532, HP1BP3, ZEB2, GLI2, GLI3, KCNIP3, MAX, ZNF738, HSF2, ANG, SETMAR, ZNF540, SOX15, ZNF496, ZNF493, CSDC2, NR2F1, ZBTB22, SOX10, AR, ELP2, ZNF280D, FOXJ2, POLR1E, LDB1, SPEN, ZFP28, ZNF333, ZNF688, PURA, ASH1L, ZNF711, ZFPM2, ZNF33B, ZNF483, SUPT3H, ZNF275, NDN, SCML1, ABI2, NFIX, ZBTB16, PRDM16, HIC1, ZNF660, ZFP36L2, TSC22D3, NPAS3, TSPYL2, NR1D1, HAND2, NR1D2, SOS1, ZNF708, CSDE1, ZNF286A, BAZ2B, CHD5, MAF, PDS5B, TBX3, AFF3, ZNF25, SNAI2, ZNF358, ZNF662, TRIM21, PKNOX2, BNC2, SP4, ZRANB2, PHF21A, DENND4A, ZBTB2, PBX1, OGG1, NFIC, NFIA, NFIB
Regulation of transcription, DNA-dependent	142	4.35E-10	*MEF2C, THRA, TCEAL1, FOXO6, PGR, ZFP92, HOXC6, ZNF181, GATA6, ZFP90, ZNF397, ZNF442, ZNF395, RARA, FOXO3B, TWIST2, ZNF43, ZNF641, SATB1, RXRA, ZHX1, MECP2, HMG20A, ARID1B, PROX1, TRERF1, FOXN3, KRBA2, ZNF197, ZNF383, PRDM2, TGFB1I1, ZIM2, ZNF519, TSHZ3, EID1, ZNF430, TSHZ2, ZNF132, TADA3, AFAP1L2, ZNF514, WT1, NR2C2, ARNT, MEIS2, HTATSF1, TFDP2, TEF, ZNF425, ZNF599, MAML3, TCF4, ASF1A, ZNF70, BMP4, ZNF529, SMAD9, KLF12, RFX7, SMAD5, TAF7, ZNF621, LMCD1, KLF15,*

			MED13L, FOXP1, SALL2, CSRNP3, DMTF1, EBF1, ATF7, ZIK1, ABL1, ENG, POU6F1, ZNF83, BACH2, ELF2, ZEB2, GLI2, KCNIP3, ZNF738, HSF2, ZNF540, SOX15, ZNF496, ZNF493, NR2F1, CSDC2, SOX10, ELP2, AR, FOXJ2, LDB1, SPEN, ZFP28, ZNF333, ZNF688, NRIP2, PURA, ZMIZ1, ZFPM2, JMJD1C, ZNF33B, ZNF483, MAPRE3, SUPT3H, ZNF275, NDN, HCFC2, NFIX, ZBTB16, PRDM16, CALCOCO1, HIC1, TSC22D3, NPAS3, NR1D1, HAND2, NR1D2, ZNF708, CSDE1, ZNF286A, PER3, MAF, KAT2B, TBX3, ZNF25, SNAI2, ZNF662, PKNOX2, ZNF862, SP4, PHF21A, DENND4A, PBX1, ID3, NFIC, APBB1, NFIA, NFIB
Regulation of RNA metabolic process	144	5.52E-10	*MEF2C, THRA, TCEAL1, FOXO6, PGR, ZFP92, HOXC6, ZNF181, GATA6, ZFP90, ZNF397, ZNF442, ZNF395, RARA, FOXO3B, TWIST2, ZNF43, ZNF641, SATB1, RXRA, ZHX1, MECP2, HMG20A, ARID1B, PROX1, TRERF1, FOXN3, KRBA2, CELF6, ZNF197, ZNF383, PRDM2, TGFB1I1, ZIM2, ZNF519, TSHZ3, EID1, ZNF430, TSHZ2, ZNF132, TADA3, AFAP1L2, ZNF514, WT1, NR2C2, ARNT, MEIS2, HTATSF1, TFDP2, TEF, ZNF425, ZNF599, MAML3, TCF4, ASF1A, ZNF70, BMP4, ZNF529, SMAD9, KLF12, RFX7, SMAD5, TAF7, ZNF621, LMCD1, KLF15, MED13L, FOXP1, SALL2, CSRNP3, DMTF1, EBF1, ATF7, ZIK1, ABL1, ENG, POU6F1, ZNF83, BACH2, ELF2, ZEB2, GLI2, KCNIP3, ZNF738, HSF2, ZNF540, SOX15, ZNF496, ZNF493, NR2F1, CSDC2, SOX10, ELP2, AR, FOXJ2, LDB1, SPEN, ZFP28, ZNF333, ZNF688, NRIP2, PURA, ZMIZ1, ZFPM2, JMJD1C, ZNF33B, ZNF483, MAPRE3, SUPT3H, ZNF275, NDN, HCFC2, NFIX, ZBTB16, PRDM16, CALCOCO1, HIC1, ZFP36L2, TSC22D3, NPAS3, NR1D1, HAND2, NR1D2, ZNF708, CSDE1, ZNF286A, PER3, MAF, KAT2B, TBX3, ZNF25, SNAI2, ZNF662, PKNOX2, ZNF862, SP4, PHF21A, DENND4A, PBX1, ID3, NFIC, APBB1, NFIA,*

			NFIB
Metal ion binding	271	2.05E-09	*ADCY2, LTBP3, ATP1B2, SCN3B, LTBP4, SNCA, ZNF781, SYT9, GTF2IRD2B, FAH, ZFP92, PGR, ZFP90, CCBE1, ZNF397, RARA, ZNF395, SCD5, CDH23, ZNF43, MATN2, NUDT16, ZNF641, CGRRF1, RXRA, ZHX1, F8, NUDT10, NUDT11, LPCAT2, PRDM8, PGM5, PRDM5, ZNF383, RYR2, NEK9, PRDM2, TGFB1I1, ADAMTS3, ADD1, ZIM2, ZNF519, ZNF132, NEK1, CACNB2, CACNB3, ZNF514, ZNF512, RPS27, CDADC1, FAHD2B, ZNF70, TRPC1, ASXL3, ZC3H13, ZNF529, CYP46A1, KLF12, PCDH10, ZNF621, SMYD4, ZNF521, KLF15, PCDH19, PRPSAP2, PCDH18, KCNJ8, HEBP1, SYTL4, ZIK1, HEPH, KLF2, PRKRIR, ZBTB33, ZCCHC24, CACHD1, PCDHA3, ZNF532, SOBP, ZEB2, GLI2, KCNIP2, GLI3, VILL, KCNIP3, SLC23A2, ZNF540, ZNF496, MBLAC2, ZNF493, NR2F1, ZBTB22, AR, PCDH11X, PCDHB4, ZNF333, ZNF688, PJA2, ZDHHC15, PJA1, NAALAD2, ZDHHC17, MAST2, ASH1L, EGFL8, ZNF33B, ZNF483, PLA2G5, ZNF275, SCAPER, PCDHB15, ZBTB16, DCHS1, PLCL2, ZNF660, CYB561D2, ZFP36L2, PLCL1, NR1D1, MORC3, DGKD, NR1D2, FAT4, HAAO, SLC4A8, ZNF286A, BAZ2B, RASA4, DTNA, CCNB1IP1, RBM20, MEX3B, ZNF25, PDZRN3, SNAI2, TRIM23, ZNF662, ZNF358, TRIM21, LRP1, PHF19, PLSCR4, FYN, BNC2, SVIL, ZNF862, PHF21A, CHN2, ZBTB2, LRP4, THRA, ZAK, APOBEC3G, TRIM52, ZXDA, ZNF181, GATA6, RNF38, ZNF442, ZNF575, RNF146, NMNAT3, ZNF594, PCDHGA11, GTF2IRD2, CDO1, TRERF1, PLCE1, KCNT2, RASGRF2, ZNF197, MFAP4, DST, SLC40A1, PRPS1, ME1, TSHZ3, TSHZ2, ZNF430, CXXC4, EXTL2, ZC3H6, NR2C2, WT1, TRIM68, TTYH2, SRR, ZNF425, ZNF599, UNKL, CACNA2D1, SETDB2, TAF3, SMG6, SYT11, LMCD1, ZNF835, TRIM61, FOXP1, EPS15, SALL2, PHF2, PHF1, ATF7, EBF1, MTR, ANTXR2, RHBDL3, ABL1,*

			ZNF83, CLSTN2, PCDHGA8, ZCWPW2, PCDHGA7, ZCWPW1, PCDHGA6, PCDHGA5, PCDHGA3, PCDHGA2, PCDHGA1, TRIM4, OSR2, ANG, SETMAR, ATP8B2, ANO6, CDK15, ZNF280D, WBSCR17, PCDH9, PCDHGB6, PFKM, NRXN1, ZFP28, PCDHGB2, PCDHGB5, PCDHGB4, ZMIZ1, ZNF711, ZFPM2, JMJD1C, PRNP, PROS1, CYP2U1, LOC375196, FKBP7, PCDHGC5, PCDHGC4, PCDHGC3, PRDM16, NAALADL1, HIC1, CPZ, ANXA6, SMOC2, AGAP11, ZNF708, ZC3H12B, ZNF609, PLCD1, EHD2, CHD5, HRC, WDFY2, B3GALT2, CYP21A2, DTX3, FAM188A, MAN1C1, SP4, ZRANB2, BMPR1B, ATP8A1
Ion binding	276	2.35E-09	*ADCY2, LTBP3, ATP1B2, SCN3B, LTBP4, SNCA, ZNF781, SYT9, GTF2IRD2B, FAH, ZFP92, PGR, ZFP90, CCBE1, ZNF397, RARA, ZNF395, SCD5, CDH23, ZNF43, MATN2, NUDT16, ZNF641, CGRRF1, RXRA, ZHX1, F8, NUDT10, NUDT11, LPCAT2, PRDM8, PGM5, PRDM5, RYR2, ZNF383, NEK9, PRDM2, TGFB1I1, ADAMTS3, ADD1, ZIM2, ZNF519, ZNF132, NEK1, CACNB2, CACNB3, ZNF514, ZNF512, RPS27, CDADC1, FAHD2B, ZNF70, TRPC1, ASXL3, ZC3H13, ZNF529, CYP46A1, KLF12, PCDH10, ZNF621, SMYD4, ZNF521, KLF15, PCDH19, PRPSAP2, PCDH18, KCNJ8, HEBP1, SYTL4, ZIK1, HEPH, KLF2, CLCN6, PRKRIR, ZBTB33, ZCCHC24, CACHD1, PCDHA3, ZNF532, SOBP, ZEB2, GLI2, KCNIP2, GLI3, VILL, KCNIP3, SLC23A2, ZNF540, ZNF496, MBLAC2, ZNF493, NR2F1, ZBTB22, AR, PCDH11X, PCDHB4, CLIC2, ZNF333, ZNF688, PJA2, ZDHHC15, PJA1, NAALAD2, ZDHHC17, MAST2, ASH1L, GLRA4, EGFL8, ZNF33B, ZNF483, PLA2G5, ZNF275, SCAPER, PCDHB15, ZBTB16, DCHS1, PLCL2, ZNF660, CYB561D2, ZFP36L2, PLCL1, NR1D1, MORC3, DGKD, NR1D2, FAT4, HAAO, SLC4A8, ZNF286A, BAZ2B, RASA4, DTNA, CCNB1IP1, RBM20, MEX3B, ZNF25, PDZRN3, SNAI2,*

			TRIM23, ZNF662, ZNF358, TRIM21, LRP1, PHF19, PLSCR4, FYN, BNC2, SVIL, ZNF862, PHF21A, CHN2, ZBTB2, LRP4, THRA, ZAK, APOBEC3G, TRIM52, ZXDA, ZNF181, GATA6, RNF38, ZNF442, ZNF575, RNF146, NMNAT3, ZNF594, PCDHGA11, GTF2IRD2, CDO1, TRERF1, PLCE1, KCNT2, RASGRF2, ZNF197, MFAP4, DST, SLC40A1, PRPS1, ME1, FXYD1, TSHZ3, TSHZ2, ZNF430, CXXC4, HEXDC, EXTL2, ZC3H6, NR2C2, WT1, TRIM68, TTYH2, SRR, ZNF425, ZNF599, UNKL, CACNA2D1, SETDB2, TAF3, SMG6, SYT11, LMCD1, ZNF835, TRIM61, FOXP1, EPS15, SALL2, PHF2, PHF1, ATF7, EBF1, MTR, ANTXR2, RHBDL3, ABL1, ZNF83, CLSTN2, PCDHGA8, ZCWPW2, PCDHGA7, ZCWPW1, PCDHGA6, PCDHGA5, PCDHGA3, PCDHGA2, PCDHGA1, TRIM4, OSR2, ANG, SETMAR, ATP8B2, ANO6, CDK15, ZNF280D, WBSCR17, PCDH9, PCDHGB6, PFKM, NRXN1, ZFP28, PCDHGB2, PCDHGB5, PCDHGB4, ZMIZ1, ZNF711, ZFPM2, JMJD1C, PRNP, PROS1, CYP2U1, LOC375196, FKBP7, PCDHGC5, PCDHGC4, PCDHGC3, PRDM16, NAALADL1, HIC1, CPZ, ANXA6, SMOC2, AGAP11, ZNF708, ZC3H12B, ZNF609, PLCD1, EHD2, CHD5, HRC, WDFY2, B3GALT2, CYP21A2, DTX3, FAM188A, MAN1C1, SP4, ZRANB2, BMPR1B, ATP8A1
Homophilic cell adhesion	26	2.79E-09	*CLSTN2, PCDHA3, PCDHGA8, PCDHB15, PCDHGC5, PCDHGA7, PCDHGC4, PCDHGA6, PCDHGA5, PCDHGC3, PCDHGA3, PCDHGA2, DCHS1, PCDHGA1, FAT4, ROBO1, CDH23, PCDHGA11, PCDH11X, PCDHB4, PCDH10, PCDH9, PCDHGB6, PCDHGB2, PCDH19, PCDHGB5, PCDH18, PCDHGB4*
Cation binding	272	3.50E-09	*ADCY2, LTBP3, ATP1B2, SCN3B, LTBP4, SNCA, ZNF781, SYT9, GTF2IRD2B, FAH, ZFP92, PGR, ZFP90, CCBE1, ZNF397, RARA, ZNF395, SCD5, CDH23, ZNF43, MATN2, NUDT16, ZNF641, CGRRF1, RXRA, ZHX1, F8,*

NUDT10, NUDT11, LPCAT2, PRDM8, PGM5, PRDM5, ZNF383, RYR2, NEK9, PRDM2, TGFB1I1, ADAMTS3, ADD1, ZIM2, ZNF519, ZNF132, NEK1, CACNB2, CACNB3, ZNF514, ZNF512, RPS27, CDADC1, FAHD2B, ZNF70, TRPC1, ASXL3, ZC3H13, ZNF529, CYP46A1, KLF12, PCDH10, ZNF621, SMYD4, ZNF521, KLF15, PCDH19, PRPSAP2, PCDH18, KCNJ8, HEBP1, SYTL4, ZIK1, HEPH, KLF2, PRKRIR, ZBTB33, ZCCHC24, CACHD1, PCDHA3, ZNF532, SOBP, ZEB2, GLI2, KCNIP2, GLI3, VILL, KCNIP3, SLC23A2, ZNF540, ZNF496, MBLAC2, ZNF493, NR2F1, ZBTB22, AR, PCDH11X, PCDHB4, ZNF333, ZNF688, PJA2, ZDHHC15, PJA1, NAALAD2, ZDHHC17, MAST2, ASH1L, EGFL8, ZNF33B, ZNF483, PLA2G5, ZNF275, SCAPER, PCDHB15, ZBTB16, DCHS1, PLCL2, ZNF660, CYB561D2, ZFP36L2, PLCL1, NR1D1, MORC3, DGKD, NR1D2, FAT4, HAAO, SLC4A8, ZNF286A, BAZ2B, RASA4, DTNA, CCNB1IP1, RBM20, MEX3B, ZNF25, PDZRN3, SNAI2, TRIM23, ZNF662, ZNF358, TRIM21, LRP1, PHF19, PLSCR4, FYN, BNC2, SVIL, ZNF862, PHF21A, CHN2, ZBTB2, LRP4, THRA, ZAK, APOBEC3G, TRIM52, ZXDA, ZNF181, GATA6, RNF38, ZNF442, ZNF575, RNF146, NMNAT3, ZNF594, PCDHGA11, GTF2IRD2, CDO1, TRERF1, PLCE1, KCNT2, RASGRF2, ZNF197, MFAP4, DST, SLC40A1, PRPS1, ME1, TSHZ3, TSHZ2, ZNF430, CXXC4, HEXDC, EXTL2, ZC3H6, NR2C2, WT1, TRIM68, TTYH2, SRR, ZNF425, ZNF599, UNKL, CACNA2D1, SETDB2, TAF3, SMG6, SYT11, LMCD1, ZNF835, TRIM61, FOXP1, EPS15, SALL2, PHF2, PHF1, ATF7, EBF1, MTR, ANTXR2, RHBDL3, ABL1, ZNF83, CLSTN2, PCDHGA8, ZCWPW2, PCDHGA7, ZCWPW1, PCDHGA6, PCDHGA5, PCDHGA3, PCDHGA2, PCDHGA1, TRIM4, OSR2, ANG, SETMAR, ATP8B2, ANO6, CDK15, ZNF280D, WBSCR17, PCDH9, PCDHGB6, PFKM, NRXN1, ZFP28, PCDHGB2,

			PCDHGB5, PCDHGB4, ZMIZ1, ZNF711, ZFPM2, JMJD1C, PRNP, PROS1, CYP2U1, LOC375196, FKBP7, PCDHGC5, PCDHGC4, PCDHGC3, PRDM16, NAALADL1, HIC1, CPZ, ANXA6, SMOC2, AGAP11, ZNF708, ZC3H12B, ZNF609, PLCD1, EHD2, CHD5, HRC, WDFY2, B3GALT2, CYP21A2, DTX3, FAM188A, MAN1C1, SP4, ZRANB2, BMPR1B, ATP8A1
Transcription factor activity	85	1.30E-07	*MEF2C, POU6F1, ZNF83, BACH2, ELF2, THRA, ZEB2, GLI2, RFXAP, GLI3, TCEAL1, FOXO6, ZXDA, HOXC6, PGR, MAX, HSF2, GATA6, ZNF397, SOX15, RARA, FOXO3B, ZNF496, TWIST2, NR2F1, SOX10, SATB1, AR, FOXJ2, RXRA, ZHX1, HMG20A, SPEN, PROX1, TRERF1, FOXN3, PURA, ZNF197, PRDM2, ZNF33B, ZNF483, ZIM2, TSHZ3, TSHZ2, ZNF132, TADA3, SCML1, NFIX, ZBTB16, NR2C2, WT1, ARNT, HIC1, ZFP36L2, TSC22D3, MEIS2, NR1D1, NR1D2, HAND2, TFDP2, TEF, TCF4, ZNF70, MAF, SMAD9, TBX3, KLF12, SMAD5, TAF7, KLF15, FOXP1, PKNOX2, SALL2, CSRNP3, PHF1, DMTF1, ATF7, SP4, EBF1, ZRANB2, PBX1, KLF2, NFIC, NFIA, NFIB*

Supplementary Table S5. Top 10 pathways enriched in high-grade serous carcinoma *versus* normal tissues

GO Term	Count	*P* value	Genes
M phase	50	1.10E-46	*KIF23, KIF22, KIFC1, XRCC2, PRC1, NEK2, TTK, AURKA, PTTG1, AURKB, CEP55, FAM83D, KIF2C, SPC25, NCAPH, CDCA8, DDX11, NCAPG, BUB1, SKA3, SKA1, CDCA5, ASPM, ERCC6L, CDCA3, TRIP13, CDK1, KIF11, MKI67, SGOL1, KIF15, CCNF, TPX2, NUF2, KIF18A, CDC20, BIRC5, NDC80, ESPL1, TACC3, RAD54L, NCAPD2, RAD51, MAD2L1, CCNB2, PLK1, CKS2, BUB1B, CIT, KPNA2*
Cell cycle phase	52	2.08E-44	*KIF23, KIFC1, KIF22, XRCC2, PRC1, TTK, AURKA, AURKB, PTTG1, GTSE1, FAM83D, KIF2C, CDCA8, DDX11, CDCA5, ASPM, CDCA3, CDK1, KIF11, CCNF, KIF15, SGOL1, TPX2, ESPL1, TACC3, RAD51, NCAPD2, MAD2L1, BUB1B, KPNA2, BLM, NEK2, CEP55, SPC25, NCAPH, NCAPG, BUB1, SKA3, SKA1, TRIP13, ERCC6L, MKI67, KIF18A, NUF2, NDC80, BIRC5, CDC20, RAD54L, CCNB2, PLK1, CKS2, CIT*
Cell cycle	63	1.84E-43	*KIF23, KIF22, KIFC1, E2F3, XRCC2, PRC1, E2F7, TTK, AURKA, PTTG1, AURKB, GTSE1, FAM83D, KIF2C, CDCA8, DDX11, FANCI, CDCA5, ASPM, CDCA3, CDK1, KIF11, SGOL1, CCNF, KIF15, TPX2, ESPL1, TACC3, NCAPD2, RAD51, KRT18, MAD2L1, BUB1B, KPNA2, CKS1B, BLM, NEK2, FOXM1, CEP55, LLGL2, SPC25, NCAPH, NCAPG, HJURP, BUB1, SKA3, SKA1, ERCC6L, TRIP13, PARD6B, MKI67, PSRC1, KIF18A, NUF2, CDC20, BIRC5, NDC80, RACGAP1, RAD54L, CCNB2, PLK1, CKS2, CIT*
Mitosis	40	8.58E-40	*KIF23, KIF22, KIFC1, NEK2, AURKA, PTTG1, CEP55, AURKB, FAM83D, KIF2C, SPC25, NCAPH, CDCA8, DDX11, NCAPG, BUB1, SKA3, SKA1, CDCA5, ASPM, ERCC6L, CDCA3, CDK1, KIF11, SGOL1, KIF15, CCNF, TPX2, NUF2, KIF18A, CDC20, BIRC5, NDC80, ESPL1,*

			NCAPD2, MAD2L1, CCNB2, PLK1, BUB1B, CIT
Nuclear division	40	8.58E-40	*KIF23, KIF22, KIFC1, NEK2, AURKA, PTTG1, CEP55, AURKB, FAM83D, KIF2C, SPC25, NCAPH, CDCA8, DDX11, NCAPG, BUB1, SKA3, SKA1, CDCA5, ASPM, ERCC6L, CDCA3, CDK1, KIF11, SGOL1, KIF15, CCNF, TPX2, NUF2, KIF18A, CDC20, BIRC5, NDC80, ESPL1, NCAPD2, MAD2L1, CCNB2, PLK1, BUB1B, CIT*
M phase of mitotic cell cycle	40	1.81E-39	*KIF23, KIF22, KIFC1, NEK2, AURKA, PTTG1, CEP55, AURKB, FAM83D, KIF2C, SPC25, NCAPH, CDCA8, DDX11, NCAPG, BUB1, SKA3, SKA1, CDCA5, ASPM, ERCC6L, CDCA3, CDK1, KIF11, SGOL1, KIF15, CCNF, TPX2, NUF2, KIF18A, CDC20, BIRC5, NDC80, ESPL1, NCAPD2, MAD2L1, CCNB2, PLK1, BUB1B, CIT*
Organelle fission	40	4.49E-39	*KIF23, KIF22, KIFC1, NEK2, AURKA, PTTG1, CEP55, AURKB, FAM83D, KIF2C, SPC25, NCAPH, CDCA8, DDX11, NCAPG, BUB1, SKA3, SKA1, CDCA5, ASPM, ERCC6L, CDCA3, CDK1, KIF11, SGOL1, KIF15, CCNF, TPX2, NUF2, KIF18A, CDC20, BIRC5, NDC80, ESPL1, NCAPD2, MAD2L1, CCNB2, PLK1, BUB1B, CIT*
Cell cycle process	53	9.36E-39	KIF23, KIFC1, KIF22, XRCC2, PRC1, TTK, AURKA, AURKB, PTTG1, GTSE1, FAM83D, KIF2C, CDCA8, *DDX11, CDCA5, ASPM, CDCA3, CDK1, KIF11, CCNF, KIF15, SGOL1, TPX2, ESPL1, TACC3, RAD51, NCAPD2, MAD2L1, BUB1B, KPNA2, BLM, NEK2, CEP55, SPC25, NCAPH, NCAPG, BUB1, SKA3, SKA1, TRIP13, ERCC6L, MKI67, KIF18A, NUF2, NDC80, BIRC5, CDC20, RACGAP1, RAD54L, CCNB2, PLK1, CKS2, CIT*
Mitotic cell cycle	45	3.16E-37	*KIF23, KIF22, KIFC1, PRC1, BLM, NEK2, TTK, AURKA, PTTG1, CEP55, AURKB, GTSE1, FAM83D, KIF2C, SPC25, NCAPH, CDCA8, DDX11, NCAPG, BUB1, SKA3, SKA1, CDCA5, ASPM, ERCC6L, CDCA3, CDK1, KIF11, SGOL1, KIF15, CCNF, TPX2, NUF2, KIF18A, CDC20, BIRC5, NDC80, ESPL1, NCAPD2, MAD2L1,*

Cell division	39	2.87E-33	*CCNB2, PLK1, BUB1B, CIT, KPNA2 KIF23, CKS1B, KIFC1, PRC1, NEK2, PTTG1, CEP55, AURKB, LLGL2, FAM83D, SPC25, NCAPH, CDCA8, NCAPG, BUB1, SKA3, SKA1, CDCA5, ASPM, ERCC6L, CDCA3, PARD6B, CDK1, KIF11, SGOL1, CCNF, NUF2, CDC20, BIRC5, NDC80, ESPL1, RACGAP1, NCAPD2, MAD2L1, CCNB2, PLK1, CKS2, BUB1B, CIT*

Supplementary Table S6. Top 10 pathways enriched in normal tissues *versus* high-grade serous carcinoma

GO Term	Count	*P* value	Genes
Blood vessel development	10	4.25E-05	*RECK, CAV1, ANG, HAND2, LEPR, CCBE1, RHOB, NR2F2,*
Vasculature development	10	5.13E-05	*RECK, CAV1, ANG, HAND2, LEPR, CCBE1, RHOB, NR2F2,*
Blood vessel morphogenesis	9	9.29E-05	*CAV1, ANG, HAND2, LEPR, CCBE1, RHOB, NR2F2, FGF2,*
Muscle organ development	9	9.29E-05	*LAMA2, POU6F1, CAV1, SVIL, MRAS, CACNA1H, CACNB*
Angiogenesis	7	5.10E-04	*ANG, HAND2, LEPR, CCBE1, RHOB, FGF2, ENG*
Chondroitin sulfate biosynthetic process	3	1.25E-03	*CSGALNACT1, CHST7, CHST3*
Chondroitin sulfate proteoglycan biosynthetic process	3	2.42E-03	*CSGALNACT1, CHST7, CHST3*
Chondroitin sulfate metabolic process	3	3.95E-03	*CSGALNACT1, CHST7, CHST3*
Negative regulation of cellular biosynthetic process	11	4.72E-03	*CAV1, TSPYL2, ANG, PPARG, ZEB2, NR2F2, FGF2, ENG,*
Extracellular structure organization	6	5.06E-03	*CSGALNACT1, RECK, PDGFRA, CACNB2, ECM2, ENG*

Supplementary Table S7. Top 10 pathways enriched in clear cell carcinoma *versus* high-grade serous carcinoma

GO Term	Count	*P* value	Genes
Wound healing	14	1.51E-06	*KNG1, B4GALT1, GNA13, NOG, ITGA2, FGG, HIF1A, HNF4A, FGA, FGB, F3, F2, IGFBP1, PAPSS2*
Regulation of cell proliferation	29	1.53E-06	*RBP4, NOG, FGFR4, IGFBP7, MITF, PNP, ITCH, ASPH, LAMB1, THPO, B4GALT1, HYAL1, COL4A3, TESC, HCLS1, ITGA2, GJB6, PTHLH, CDKN1A, CTH, HIF1A, HNF4A, COG8, SSTR1, DLX5, F3, SCIN, LAMC1, TOB2*
Coagulation	10	8.10E-06	*GNA13, KNG1, FGG, HNF4A, FGA, FGB, F3, F2, ITGA2, PAPSS2*
Blood coagulation	10	8.10E-06	*GNA13, KNG1, FGG, HNF4A, FGA, FGB, F3, F2, ITGA2, PAPSS2*
Hemostasis	10	1.29E-05	*GNA13, KNG1, FGG, HNF4A, FGA, FGB, F3, F2, ITGA2, PAPSS2*
Negative regulation of cell proliferation	17	2.43E-05	*B4GALT1, RBP4, COL4A3, HYAL1, TESC, NOG, IGFBP7, GJB6, PTHLH, CTH, CDKN1A, HNF4A, SSTR1, SCIN, ASPH, ITCH, TOB2*
Regulation of body fluid levels	10	1.06E-04	*GNA13, KNG1, FGG, HNF4A, FGA, FGB, F3, F2, ITGA2, PAPSS2*
Response to wounding	19	2.34E-04	*B4GALT1, GNA13, KNG1, NOG, ITGA2, FGG, HIF1A, FGA, HNF4A, FGB, F3, F2, MGLL, RTN4RL2, LBP, IGFBP1, ITCH, PAPSS2, BLNK*
Cell surface binding	5	6.47E-04	*FGG, FGA, FGB, F3, LBP*
Platelet activation	5	7.98E-04	*GNA13, FGG, FGA, FGB, F2*

Supplementary Table S8. Top 10 pathways enriched in high-grade serous carcinoma *versus* clear cell carcinoma

GO Term	Count	*P* value	Genes
Transcription	27	9.17E-04	*ZNF519, ZNF827, CTCFL, NFIX, MEIS1, TCF7L2, FOXO6, WT1, ZNF512, ZFP92, NPAS3, PPP1R1B, PER3, BHLHE41, BAZ2B, PHOX2A, SSBP3, KLF12, ESR1, SPEN, ZNF334, EYA2, CDCA7L, PBX1, ZNF99, HDAC7, NFIB*
Regulation of transcription	31	1.05E-03	*ZNF519, CRABP2, ZNF827, CTCFL, NFIX, MEIS1, TCF7L2, WT1, ZNF512, FOXO6, ZFP92, NPAS3, PER3, BAZ2B, BHLHE41, PHOX2A, SSBP3, KLF12, EMX2, ESR1, SPEN, ABCG4, ZNF334, PRKCQ, EYA2, CDCA7L, PBX1, LRCH4, ZNF99, HDAC7, NFIB*
DNA binding	26	1.83E-03	*ZNF519, ZNF827, ABI2, CTCFL, NFIX, MEIS1, TCF7L2, WT1, ZNF512, FOXO6, ZFP92, NPAS3, BHLHE41, BAZ2B, PHOX2A, SSBP3, KLF12, CBL, EMX2, ESR1, SPEN, ZNF334, H2AFY2, PBX1, ZNF99, NFIB*
Neuron differentiation	10	2.50E-03	*PHOX2A, PRKCQ, GPC2, RET, DRD2, CLIC5, EMX2, NTNG1, ABI2, IGSF9*
Transcription regulator activity	19	3.17E-03	*PHOX2A, CUTA, SSBP3, KLF12, CBL, EMX2, ESR1, CTCFL, NFIX, SPEN, MEIS1, TCF7L2, FOXO6, WT1, NPAS3, PBX1, LRCH4, BHLHE41, NFIB*
Embryonic morphogenesis	8	4.34E-03	*RET, EYA2, CLIC5, VANGL2, CRABP2, AMOT, PBX1, TCF7L2*
Transcription factor activity	14	5.08E-03	*PHOX2A, KLF12, EMX2, CBL, ESR1, NFIX, SPEN, TCF7L2, MEIS1, FOXO6, WT1, PBX1, BHLHE41, NFIB*
Regulation of RNA metabolic process	21	1.45E-02	*PHOX2A, ZNF519, KLF12, CRABP2, EMX2, ESR1, NFIX, SPEN, MEIS1, TCF7L2, FOXO6, WT1, ZNF334, ZFP92, NPAS3, RBM38, PBX1, PER3, BHLHE41, ZNF99, NFIB*
Epithelium development	6	1.77E-02	*RET, VANGL2, TGM1, PBX1, CDSN, WT1*
Regulation of transcription, DNA-dependent	20	2.28E-02	*PHOX2A, ZNF519, KLF12, CRABP2, EMX2, ESR1, NFIX, SPEN, MEIS1, TCF7L2, FOXO6, WT1, ZNF334, ZFP92, NPAS3, PBX1, PER3, BHLHE41, ZNF99, NFIB*

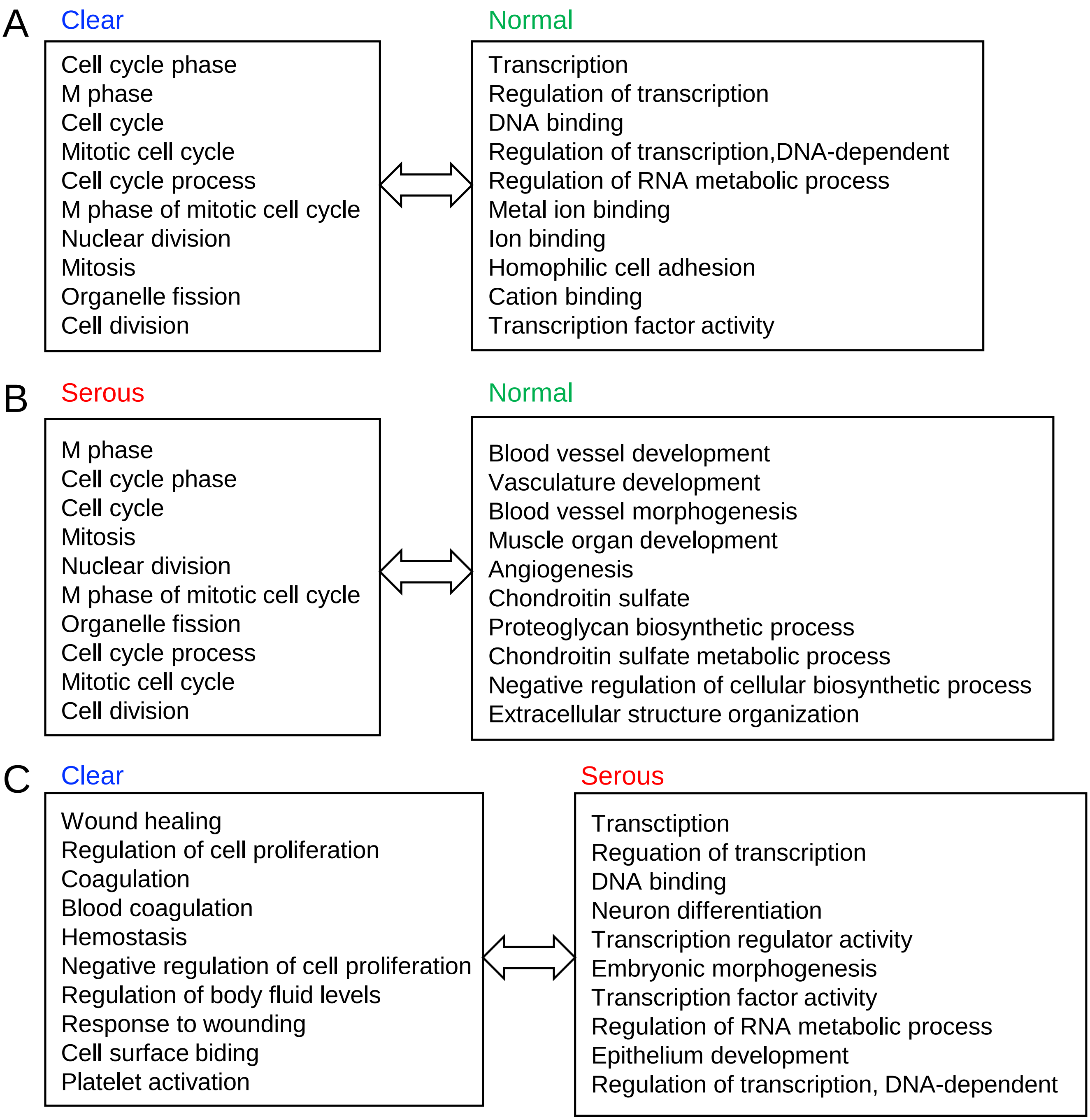

Supplementary Figure S1. Pathway enrichment analysis using PC1-contributing genes in PCA. Principal component analysis (PCA) was performed based on the expression level represented by log2RPKM value for each RefSeq CCC, HGSC, and normal tissues. DAVID Bioinformatics Resources 6.7 showed differentially expressed genes in pathways and clusters of functionally related genes in clear cell carcinoma compared to normal tissues (A), in serous carcinoma compared to normal tissues (B), and in clear cell carcinoma compared to serous carcinoma (C).

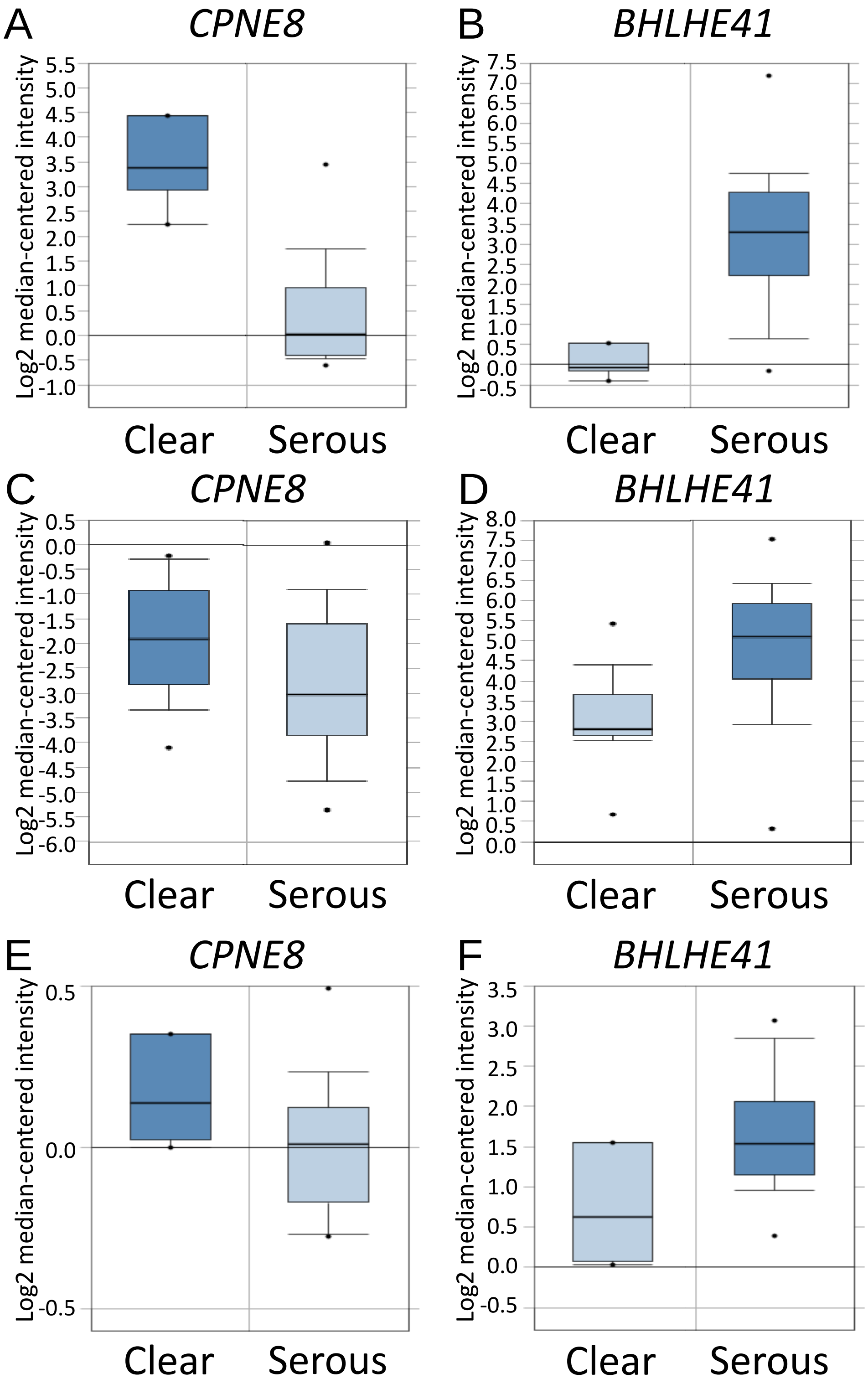

Supplementary Figure S2. Gene expression levels of *CPNE8* and *BHLHE41* based on Oncomine. (A) *CPNE8* was expressed higher in clear cell carcinoma (n = 6) than in serous carcinoma (n = 71) among Meyniel Ovarian Statistics (n = 140). (B) *BHLHE41* was expressed higher in serous carcinoma than in clear cell carcinoma among the same dataset with (A). (C) *CPNE8* was expressed higher in clear cell carcinoma (n = 13) than in serous carcinoma (n = 79) among Bittner Ovarian Statistics (n = 241). (D) *BHLHE41* was expressed higher in serous carcinoma than in clear cell carcinoma among the same dataset with (C). (E) *CPNE8* was expressed higher in clear cell carcinoma (n = 7) than in serous carcinoma (n = 20) among Lu Ovarian Statistics (n = 50). (F) *BHLHE41* was expressed higher in serous carcinoma than in clear cell carcinoma among the same dataset with (E).

A

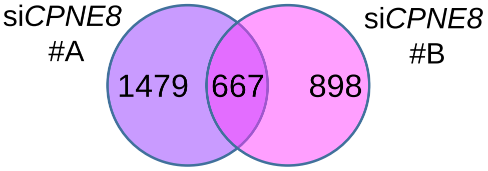

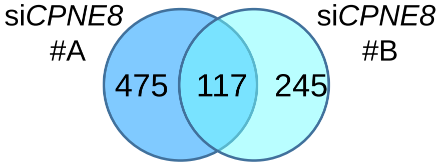

Top 6 pathways of upregulated genes by si*CPNE8*

GO Term	Count	*P* value	Genes (Selected)
Protein kinase cascade	22	8.9E-04	*SRPK2, CRYAB, ERBB2, TAOK3, STK17B*
Ion binding	147	1.0E-03	*APOBEC2, CYP24A1, ADCY2, DZIP1*
Cation binding	145	1.1E-03	*APOBEC2, CYP24A1, ADCY2, DZIP1, DBF4*
Carboxylic acid transport	12	1.9E-03	*SLC25A20, SLC6A8, SLC7A2, FOLR1, PPARG*
Organic acid transport	12	2.0E-03	*SLC25A20, SLC6A8, SLC7A2, FOLR1, PPARG*
Metal ion binding	142	2.1E-03	*APOBEC2, CYP24A1, ADCY2, DZIP1, DBF4*

Top 6 pathways of downregulated genes by si*CPNE8*

GO Term	Count	*P* value	Genes (Selected)
Response to wounding	15	3.6E-07	*PLAT, CIITA, CXCL1, NOG, TNF*
Wound healing	9	5.5E-06	*PLAT, NOG, HNF4A, EREG, HBEGF*
Regulation of cell proliferation	16	7.7E-06	*CXCL1, IRS2, NOG, TNF, EDN1*
Regulation of gene-specific transcription	7	6.1E-05	*CIITA, KDM1A, TNF, HNF4A, ETS1*
Negative regulation of transport	7	6.3E-05	*INHBB, IRS2, TNF, HNF4A, EDN1*
Regulation of body fluid levels	7	8.1E-05	*PLAT, KCNMA1, HNF4A, EDN1, CD40*

B

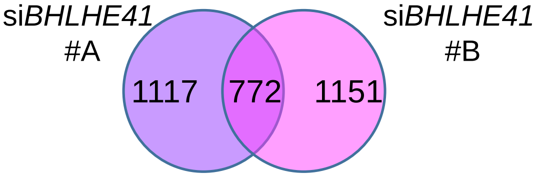

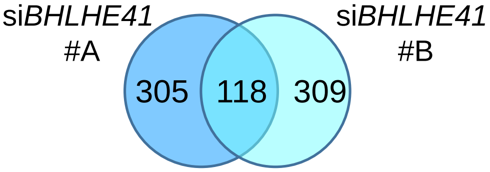

Top 6 pathways of upregulated genes by si*BHLHE41*

GO Term	Count	*P* value	Genes (Selected)
Cation binding	176	9.8E-05	*LTBP1, CHIA, PTGS1, PDLIM1, RNF215*
Metal ion binding	173	1.9E-04	*LTBP1, PTGS1, PDLIM1, RNF215, ZNF638*
Ion binding	176	2.3E-04	*LTBP1, CHIA, PTGS1, PDLIM1, RNF215*
Regulation of neurogenesis	15	7.9E-04	*NBN, PLXNB1, LIMK1, TP53, RTN4R*
Regulation of neuron differentiation	13	1.0E-03	*NBN, PLXNB1, LIMK1, RTN4R, SOX5*
Regulation of nervous system development	16	1.1E-03	*NBN, PLXNB1, LIMK1, RTN4R, SOX5*

Top 6 pathways of downregulated genes by si*BHLHE41*

GO Term	Count	*P* value	Genes (Selected)
Response to wounding	17	1.3E-09	*IRAK2, IL6, TNF, CCL2, S100A8*
Inflammatory response	14	2.0E-09	*IRAK2, IL6, TNF, CCL2, S100A8*
Defense response	16	7.9E-08	*IRAK2, IL6, TNF, CCL2, C5AR1*
Cytokine-mediated signaling pathway	6	1.7E-05	*IRAK2, IL6, TNF, CCL2, LRP8*
Chemotaxis	7	8.9E-05	*IL6, C5AR1, CCL2, SAA2, CCL20*
Taxis	7	8.9E-05	*IL6, C5AR1, CCL2, SAA2, CCL20*

Supplementary Figure S3. Up- and down-regulated pathway genes by siRNAs targeting *CPNE8* and *BHLHE41*. (A) Venn diagram showing the number of overlapping upregulated (>1.5-fold, left panel) and downregulated (<0.66-fold, right panel) microarray probes in RMG1 cells after treatment of si*CPNE8* #A and #B compared with siControl. Pathway analysis using the overlapping genes shows high enrichment of ion-related and neuron-related pathways in upregulated genes, whereas high enrichment of cytokines and inflammatory pathways in downregulated genes. (B) Similar microarray analysis was performed as in (A) except that OVCAR3 cells and si*BHLHE41* #A and #B were used. Pathway analysis shows high enrichment of protein kinase and ion-related pathways in upregulated genes, whereas high enrichment of wound response and cell proliferation pathways in downregulated genes.
